# The exercise hormone irisin has neuroprotective effects in a mouse model of multiple sclerosis

**DOI:** 10.1038/s42255-026-01527-7

**Published:** 2026-05-21

**Authors:** Sina C. Rosenkranz, Joana F. da Rocha, Luis Moreira, Pius Schlachter, Jasmina Bier, Kaela Healy, Daniela Neves Silva, Mohamed Ariff Iqbal, Marjan Gharagozloo, Yueyue Xiong, Matthew A. Murphy, Helena C. Lichtenfeld, Lukas Raich, Michaela Schweizer, Asude Ertaş, Marcel S. Woo, Vanessa Vieira, Samuel E. Honeycutt, James P. White, Gregory A. Wyant, Manuel A. Friese, Peter A. Calabresi, Ruxandra F. Sîrbulescu, Christiane D. Wrann

**Affiliations:** 1 Cardiovascular Research Center, Massachusetts General Hospital and Harvard Medical School, Boston, MA USA; 2 Institute of Neuroimmunology and Multiple Sclerosis, University Medical Center Hamburg-Eppendorf, Hamburg, Germany; 3 Department of Neurology, Johns Hopkins University, School of Medicine, Baltimore, MD USA; 4 Vaccine and Immunotherapy Center, Massachusetts General Hospital, Harvard Medical School, Boston, MA USA; 5 Core Facility Morphology and Electron Microscopy, ZMNH, University Medical Center Hamburg-Eppendorf, Hamburg, Germany; 6 Department of Medicine, Duke University School of Medicine, Durham, NC USA; 7 Department of Neurology, Mass General Brigham, Harvard Medical School, Boston, MA USA; 8 McCance Center for Brain Health, Massachusetts General Hospital, Boston, MA USA

**Keywords:** Diseases of the nervous system, Metabolism

## Abstract

Aerobic exercise is a disease-modifying intervention in multiple sclerosis (MS) that ameliorates several progressive neurological symptoms in people with MS. Here we show that the exercise hormone irisin mediates neuroprotective effects of exercise in the experimental autoimmune encephalomyelitis (EAE) mouse model of MS. We demonstrate that voluntary free-wheel running exercise protects against inflammation-induced neurodegeneration in EAE, but these neuroprotective effects are abrogated in mice lacking *Fndc5*/irisin. Peripheral delivery of irisin increases irisin plasma levels and reduces both clinical symptoms and neuronal loss in EAE. Although peripheral irisin does not alter peripheral and central immune responses in EAE, it induces a direct neuroprotective gene programme in spinal cord neurons and preserves synapses and mitochondrial activity, probably through direct binding to motor neurons. Taken together, these findings suggest that irisin induction in response to exercise confers direct neuroprotective effects in an inflammation-driven neurodegenerative condition, making it an attractive therapeutic candidate for MS.

## Abstract (web summary)

Exercise-mediated neuroprotection in a mouse model of multiple sclerosis is reduced in mice lacking the irisin-encoding gene, *Fndc5*, and can be recapitulated by peripheral administration of irisin.

## Main

Aerobic exercise has been considered a disease-modifying intervention in multiple sclerosis (MS), as it not only improves the physical fitness of people with MS but also ameliorates progressive neurological symptoms^1,2^. The exact mechanisms are unknown, and currently, no drug is available that harnesses these therapeutic effects. The exercise hormone irisin, the secreted form of fibronectin type III domain-containing protein 5 (FNDC5), is a prime candidate to mediate part of the neuroprotective effects of exercise in MS. Exercise increases *Fndc5* gene expression in skeletal muscle and the hippocampus, as well as irisin protein levels in plasma. Irisin consists of 112 amino acids, is glycosylated and is 100% conserved between mouse and humans and can cross the blood–brain barrier. Recent work proposed that irisin acts via αV/β5 integrin receptors, although the cell type and exact cellular downstream mechanism remain to be determined^1–3^. Some work suggests direct effects on astrocytes or microglia, while other research indicates direct effects on neurons^3–6^. Irisin is a key regulator of cognitive function in exercise but also in ageing and Alzheimer’s disease^3,7–9^. Furthermore, irisin ameliorates disease outcomes in preclinical models of Alzheimer’s disease and Parkinson’s disease and in a human cellular ‘Alzheimer’s-in-a dish model’^3–6^.

Here, we show that voluntary free-wheel running exercise protected against inflammation-induced neurodegeneration in experimental autoimmune encephalomyelitis (EAE), an established mouse model of MS. Genetic loss of *Fndc5/*irisin abrogated the neuroprotective effects of running exercise in EAE. Peripheral delivery of irisin, resulting in higher irisin plasma levels, improved hippocampal and spinal cord clinical symptoms of EAE and led to a decrease in neuronal loss in the spinal cord, hippocampus and retina. Interestingly, peripheral irisin did not alter peripheral and central immune responses but rather induced a neuroprotective gene programme in spinal cord neurons, mainly in genes associated with synaptic and mitochondrial function. Treatment with irisin and voluntary running rescued synapse loss and enhanced mitochondrial complex IV activity in the ventral horn during EAE, probably through direct binding to motor neurons. Taken together, these findings suggest that exercise-induced irisin confers direct neuroprotective effects in an inflammation-driven neurodegenerative condition, making it an attractive therapeutic target for MS.

## Genetic loss of *Fndc5/*irisin abrogates neuroprotective effects of running exercise in EAE

Neurological impairment caused by MS is a substantial and growing health burden. Persistent inflammation leads to continuous neurodegeneration and worsening neurological disability over time^10^. Although immunosuppressive drugs effectively modulate central nervous system (CNS)-infiltrating immune cells in MS, there are currently no approved neuroprotective therapies that directly target neurons to halt inflammation-induced neurodegeneration in MS^11^. The absence of direct neuroprotective therapies highlights a strong clinical need^12^. Physical exercise is proposed as a potential disease-modifying intervention in MS, offering clear benefits to physical abilities such as strength, endurance and balance, while also helping to alleviate progressive MS symptoms^2,13,14^. However, it is still uncertain whether regular exercise can mitigate inflammation-induced neurodegeneration. Furthermore, a direct link between exercise-induced mechanisms and disease-modifying effects in MS has yet to be established^15,16^. Although some studies have shown beneficial effects of exercise on MS rodent models, most of the interventions were associated with stress (for example, swimming exercise or forced treadmill running)^17^. Stress represents a confounder because of its well-known impact on immune-cell activation and on disease severity in EAE. Furthermore, previous studies mainly focused on the anti-inflammatory effects of exercise, not the potential neuroprotective effects^18,19^ and none has yet defined a molecular mediator for the observed disease-modifying effects^15^.

**Fig. 1:**
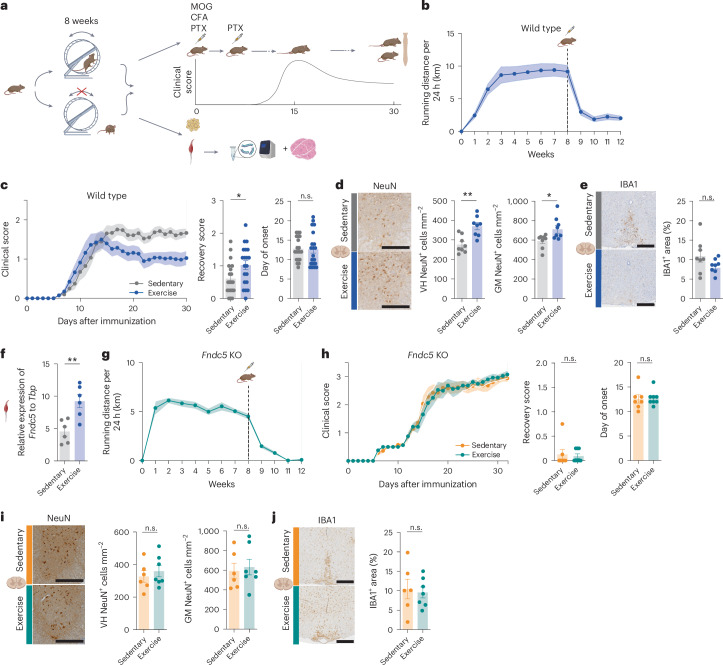
Running exercise-induced neuroprotection in EAE is abolished in *Fndc5*-KO mice. **a**, WT or *Fndc5*-KO mice had voluntary access to running wheels that were blocked (sedentary) or not (exercise). After 8 weeks, mice were either used for tissue collection or for EAE induction. **b**, Running activity of WT EAE mice (*n* = 20). **c**, Clinical scores, recovery score (*P* = 0.0275) and day of onset of WT sedentary (*n* = 20) and exercise (*n* = 20) mice undergoing EAE. **d**, Representative images and analysis of histopathological stainings of surviving NeuN^+^ neurons in spinal cord ventral horn (VH; *P* = 0.0015) and complete grey matter (GM; *P* = 0.0238) (WT sedentary *n* = 8, WT exercise *n* = 8) in chronic phase of EAE. Scale bar, 400 µm. **e**, Representative images and analysis of histopathological stainings of IBA1^+^ microglia and CNS macrophages in spinal cord sections (WT sedentary *n* = 8, WT exercise *n* = 8) in chronic phase of EAE. Scale bar, 200 µm. **f**, qRT–PCR mRNA expression levels of *Fndc5* in the gastrocnemius muscle (*P* = 0.0037) (WT sedentary *n* = 6, WT exercise *n* = 6). **g**, Running activity of *Fndc5*-KO EAE mice (*n* = 8). **h**, Clinical scores, recovery score and day of onset of *Fndc5*-KO sedentary (*n* = 7) and exercise (*n* = 8) mice undergoing EAE. **i**, Representative images and analysis of histopathological stainings of surviving NeuN^+^ neurons in spinal cord VH and complete GM (*Fndc5*-KO sedentary *n* = 6, *Fndc5*-KO exercise *n* = 7) in chronic phase of EAE. Scale bar, 400 µm. **j**, Representative images and analysis of histopathological stainings of IBA1^+^ microglia and CNS macrophages in spinal cord sections (*Fndc5*-KO sedentary *n* = 6, *Fndc5*-KO exercise *n* = 7) in chronic phase of EAE. Scale bar, 200 µm. Data are shown as mean ± s.e.m. of biological independent samples. Statistical analysis was performed using a two-tailed Mann–Whitney *U* test (**c** and **h**, recovery score), unpaired two-tailed Student’s *t*-test (**c** and **h**, day of onset and **d**–**f**, **i** and **j**). *P < 0.05, **P < 0.01. Panel **a** created in BioRender; Rosenkranz, S. https://biorender.com/e7pee06 (2025). Icons in **b**, **d**–**f**, **i**, **j** created in BioRender; Rosenkranz, S. https://biorender.com/b9z8gg0 (2025). Source data

**Extended Data Fig. 1:**
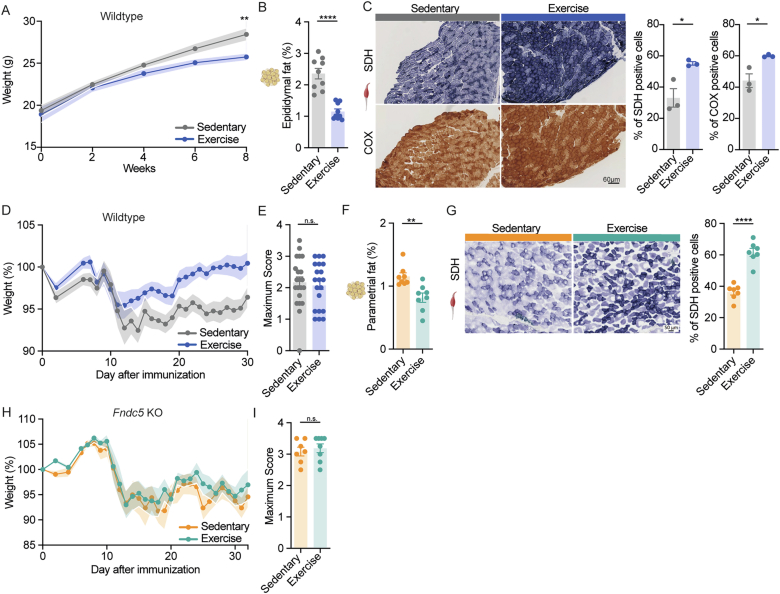
Running exercise induced neuroprotection in experimental autoimmune encephalomyelitis (EAE) is abolished in *Fndc5* Knockout (KO) mice. Single-housed wild-type (WT) mice had voluntary access to a running wheel that was blocked (sedentary) or not (running). After eight weeks, mice were either used for analysis of the gastrocnemius muscle and epididymal fat pads or for EAE induction. **a**. Body weight of sedentary (n = 9) and running (n = 9) wildtype mice. Week 8: P = 0.0026. **b**. Percentage of weight of epididymal fat pads in relation to body weight of sedentary (n = 9) and running (n = 9) wildtype mice *P* = < 0.0001. **c**. Representative images and analysis of type 1 aerobic muscle fibers represented by cytochrome c oxidase (COX) *P* = 0.0226 and succinate dehydrogenase (SDH) *P* = 0.0217 staining (n/group = 3), Scale bar: 60 µm. **d**. Percentage of body weight change of sedentary (n = 20) and running (n = 20) mice undergoing EAE *P* = 0.1081. **e**. Maximum score of sedentary (n = 20) and running (n = 20) mice undergoing EAE. **f**. Percentage of weight of parametrial fat pads in relation to body weight *P* = 0.0026 of sedentary (n = 8) and voluntary wheel running (n = 8) *Fndc5* KO mice. **g**. Analysis of type 1 aerobic muscle fibers represented by succinate dehydrogenase (SDH) staining *P* = < 0.0001 (n/group = 7). **h**. Percentage of body weight change of sedentary (n = 7) and voluntary wheel running (n = 8) *Fndc5* KO mice undergoing EAE. **i**. Maximum score of sedentary (n = 7) and voluntary wheel running (n = 8) *Fndc5* KO mice undergoing EAE. Data as mean ± SEM of biological independent samples. Statistical analysis was performed with Repeated-Measures-Two-Way ANOVA with Tukey’s post hoc test (Time x voluntary wheel running: p = 0.035, Time: p < 0.0001, voluntary wheel running: p = 0.0914, Subject: p < 0.0001) (**a**), unpaired two-tailed Student’s t-test (**b** and **c, f, g**), two-tailed Mann–Whitney U test of the area under the curve (**d** and **h**) and two tailed MannWhitney U test (**e** and **i**). *p < 0.05, **p < 0.01, ***p < 0.001. Icons in **b**, **c**, **f**, **g** created in BioRender; Rosenkranz, S. https://biorender.com/b9z8gg0 (2025). Source data

We therefore investigated the disease-modifying effects of voluntary free-wheel running exercise in the EAE model of MS. To induce EAE, mice receive an active immunization with the myelin oligodendrocyte glycoprotein (MOG_35–55_) peptide and typically develop symptoms 7–10 days after immunization, reaching peak symptom severity around days 15–18 owing to extensive immune-cell infiltration (acute phase), followed by a chronic phase with persistent disability associated with neuronal loss. Wild-type (WT) mice were randomly assigned to a cage with voluntary access to a blocked (sedentary) or unblocked (voluntary wheel running) running wheel. Of note, for this study, we used running and exercise interchangeably for ‘voluntary free-wheel running’. After 8 weeks of voluntary wheel running, EAE was induced (Fig. 1a). On average, the mice ran 7.8 km per day (± s.e.m. of 0.79; Fig. 1b) before EAE induction. This running intervention was accompanied by the expected effects of lower body weight gain, lower epididymal fat pad weight and higher oxidative capacity in muscle cells, suggestive of a muscle fibre type switch towards the high oxidative capacity type 1 muscle fibres, compared with sedentary mice (Extended Data Fig. 1a–c). The voluntary wheel running resulted in improved recovery from EAE peak symptoms (Fig. 1c and Extended Data Fig. 1d,e). Furthermore, neuronal loss, measured by the numbers of neuronal nuclei (NeuN)^+^ cells in the ventral horn and grey matter of the spinal cord, was reduced in runners compared with sedentary control mice (Fig. 1d). In addition, there was a trend towards a reduced number of ionized calcium binding adaptor molecule (IBA1)^+^ microglia and macrophages in the spinal cord with running (Fig. 1e; *P* = 0.0895). A candidate molecule that could convey the benefits of exercise in MS is the exercise-induced myokine irisin^7,8^. *Fndc5* mRNA, which encodes the precursor protein for irisin, was significantly increased in the gastrocnemius muscle with running (Fig. 1f).

To investigate whether irisin is required for the neuroprotective effects of running in EAE, global *Fndc5* knockout (KO) mice, which lack irisin^3^, underwent the same running intervention followed by EAE induction (Fig. 1a). *Fndc5*-KO mice develop normally and, importantly, do not show differences in running capacity^3^. *Fndc5*-KO mice ran an average of 5.4 km per day (± s.e.m. 0.33) (Fig. 1g). Voluntary wheel running was accompanied by expected effects on the percentage of body fat and higher oxidative capacity in muscle cells compared with sedentary *Fndc5*-KO mice (Extended Data Fig. 1f,g). Despite running, the exercise-induced rescue of clinical symptoms was completely abolished in the *Fndc5*-KO mice (Fig. 1h and Extended Data Fig. 1h,i). Furthermore, there was no significant difference in EAE-induced neuronal loss in the ventral horn and grey matter (Fig. 1i) and microglial and macrophage activation (Fig. 1j) in *Fndc5*-KO runners and sedentary mice. Taken together, these results show that irisin is required for the neuroprotective effects of voluntary running in the EAE model.

## Peripheral irisin rescues neuronal loss and improves disease outcomes in EAE

**Fig. 2:**
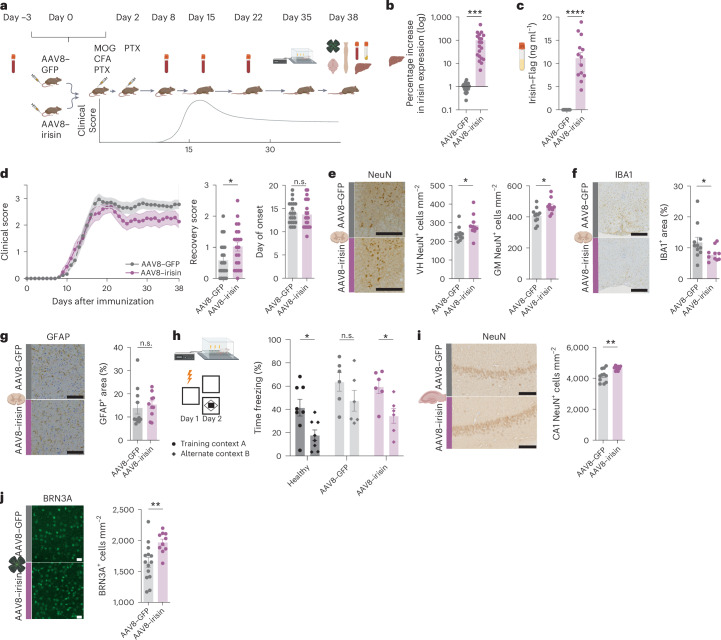
Peripheral irisin improves disease outcomes in EAE and rescues neuronal loss. **a**, WT mice were injected with AAV8–GFP or AAV8–irisin–Flag via the tail vein with subsequent EAE induction, longitudinal blood draws, hippocampal behavioural assessment and tissue collection at the end of the experiment. **b**,**c**, qRT–PCR mRNA expression levels of *irisin* in the liver (*P* = 0.0002) (AAV8–GFP *n* = 21, AAV8–irisin–Flag *n* = 20) (**b**) and in the plasma measured by ELISA at the end of EAE (*P* = < 0.0001) (AAV8–GFP *n* = 15, AAV8–irisin–Flag *n* = 13) (**c**). **d**, Mean clinical scores, recovery score (*P* = 0.0256) and day of onset of AAV8–GFP (*n* = 21) and AAV8–irisin–Flag (*n* = 21) mice undergoing EAE. **e**, Representative images and analysis of histopathological stainings of surviving NeuN^+^ neurons in spinal cord ventral horn (VH; *P* = 0.0397) and complete grey matter (GM; *P* = 0.0106) (AAV8–GFP *n* = 11, AAV8–irisin–Flag *n* = 10) in the chronic phase. Scale bar, 400 µm. **f**,**g**, Representative images and analysis of histopathological stainings of microglia and CNS macrophages (IBA1+; *P* = 0.0462) (**f**) and astrocytes (GFAP+) (**g**) in spinal cord sections (AAV8–GFP *n* = 11, AAV8–irisin–Flag *n* = 10). **h**, CFC test for healthy (*P* = 0.0107), GFP (*P* = 0.2275) and irisin (*P* = 0.0363) (healthy *n* = 8, AAV8–GFP *n* = 6, AAV8–irisin–Flag *n* = 6). **i**, Representative images and quantification of histopathological stainings of surviving NeuN^+^ neurons in the CA1 region of the hippocampus (*P* = 0.0016) (AAV8–GFP *n* = 11, AAV8–irisin–Flag *n* = 10) in the chronic phase of EAE. Scale bar, 100 µm. **j**, Representative images and analysis of immunohistochemistry stainings of surviving BRN3A^+^ RGCs (*P* = 0.0063) (AAV8–GFP *n* = 14, AAV8–irisin–Flag *n* = 10) in the chronic phase of EAE. Scale bar, 20 µm. Data are shown as mean ± s.e.m. of biological independent samples. Statistical analysis was performed using an unpaired two-tailed Student’s *t*-test (**b**–**d** day of onset, **e**–**g**, **i** and **j**), two-tailed Mann–Whitney *U* test (**d** recovery score), and one-way ANOVA with uncorrected Fisher’s LSD (**h**). *P < 0.05, **P < 0.01, ***P < 0.001, ****P < 0.0001. Panel **a** created in BioRender; Rosenkranz, S. https://biorender.com/n60s795 (2025). Icons in **b**, **c**, **e**–**j** created in BioRender; Rosenkranz, S. https://biorender.com/b9z8gg0 (2025). Source data

**Extended Data Fig. 2:**
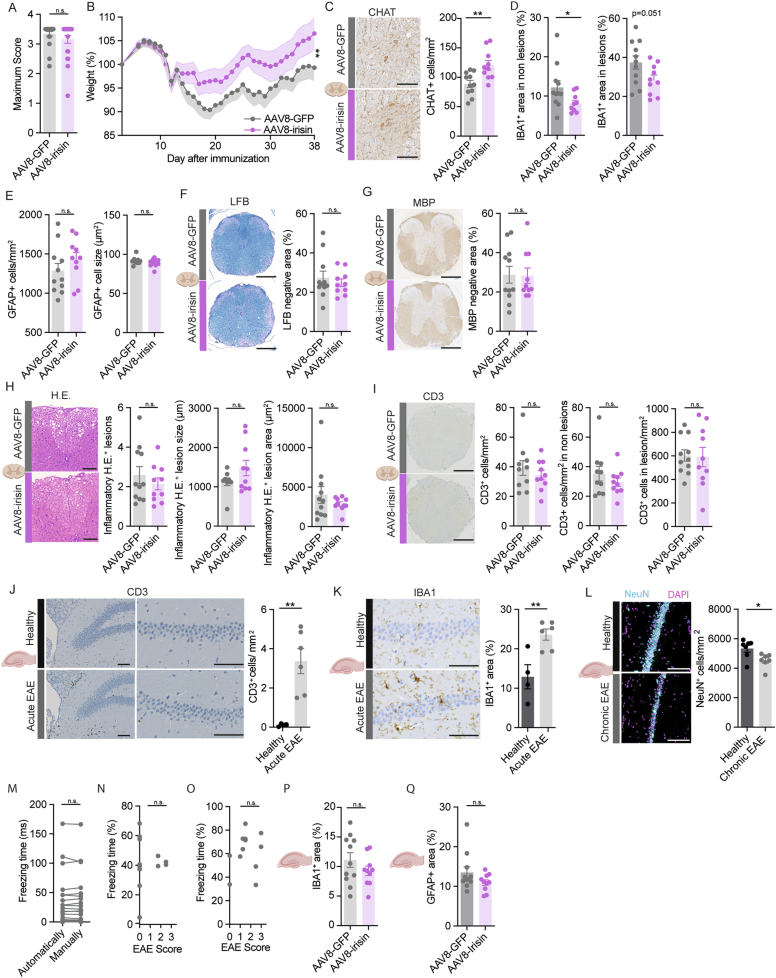
Peripheral irisin improves disease outcomes in EAE and rescues neuronal loss. WT mice were injected with AAV8-GFP or AAV8-irisin-FLAG via the tail vein with subsequent EAE induction, longitudinal blood draws, hippocampal behavioral assessment, and tissue collection at the end of the experiment. **a-b**. Maximum score (**a**) and percentage of body weight change *P* = 0.0067 (**b**) of AAV8-GFP (n = 21) and AAV8-irisin-FLAG (n = 21) mice undergoing EAE. **c-e**. Representative images and analysis of histopathological stainings of surviving CHAT^+^ motor neurons *P* = 0.0043 (**c**), area covered by IBA1^+^ microglia and CNS macrophages in non-lesion *P* = 0.0441 and in lesion areas *P* = 0.0512 (**d**) and numbers and cell size of GFAP^+^ asttrocytes in spinal cord ventral horn of AAV8-GFP (n = 11, n = 10 for GFAP^+^ cell size) and AAV8-irisin-FLAG (n = 10) mice in chronic phase of EAE, Scale bar 100 µm. **f-i**. Representative images and analysis of myelinated area by Luxol fast blue staining (**f**), Myelin basic protein (**g**), inflammatory lesion numbers, size and area by H.E. staining (**h**), infiltrating CD3^+^ T-cells in complete, lesion and non-lesion areas (**I**) of spinal cords sections of AAV8-GFP (n = 11, n = 10 for non-lesion area of CD3^+^ T-cells, n = 9 for inflammatory lesion size) and AAV8-irisin-FLAG (n = 10) mice in chronic phase. Scale bars 400 µm (**f**, **g, i**) and 200 µm **(h)**. **j-k**. Representative images and analysis of infiltrating CD3^+^ T-cells *P* = 0.0037 (**j**) and area covered by IBA1^+^ microglia and CNS macrophages *P* = 0.0073 in hippocampal sections of healthy WT (n = 4) or EAE WT (n = 6) mice in acute phase of EAE, Scale bars 100 µm. **l**. Representative images and analysis of histopathological stainings of surviving NeuN^+^ neurons in the CA1 region of the hippocampus of healthy WT (n = 7) or EAE WT (n = 7) mice *P* = 0.0202 in chronic phase of EAE, Scale bars 50 µm. **m**. Freezing times measured manually by the experimenter compared with freezing times measured automatically by the AnyMaze Software of WT EAE mice in chronic phase (n = 4). **n**. Correlation of clinical score of EAE WT mice (n = 4) compared to healthy WT mice (n = 8) used in the CFC assay and their freezing time in training context (**a**). **o**. Correlation of clinical score of AAV8-GFP and AAV8-irisin-FLAG mice (n = 12) used in the CFC assay and their freezing time in conditioning context. **p** and **q**. Analysis of histopathological stainings of IBA1^+^ microglia and CNS macrophages (**p**) and GFAP^+^-astrocytes (**q**) in hippocampal CA1 sections (AAV8-GFP, n = 11, AAV8-irisin-FLAG, n = 10) in chronic phase. Data as mean ± SEM of biological independent samples. Statistical analysis was performed with a two-tailed Mann-Whitney U test (**a**), two-tailed Mann–Whitney U test of the area under the curve (**b**), unpaired two-tailed Student’s t-test (**c-l, p, q**), paired two tailed Student’s t-test (**m**) and Pearson’s r (**n, o**). *p < 0.05, **p < 0.01. Icons in **c**, **f**–**l**, **p**, **q** created in BioRender; Rosenkranz, S. https://biorender.com/b9z8gg0 (2025). Source data

We next investigated whether irisin confers neuroprotection in EAE. Interestingly, exercise induces irisin serum levels not only in people with relapsing–remitting MS but also in people with progressive MS with higher disability scores^20,21^. Therefore, we evaluated the disease-modifying effects of peripherally delivered irisin. WT mice received AAV8–irisin–Flag (irisin-treated) or AAV8–GFP (control mice) via tail-vein injections on the day of immunization. This regimen ensured maximal irisin expression around the onset of the first neurological symptoms (8 days after induction of EAE), as full expression of AAV8 requires 10–14 days. This strategy allowed us to assess the therapeutic potential of irisin after disease onset (Fig. 2a). Tail vein-delivered AAV8–irisin–Flag expressed in the liver resulted in increased irisin plasma concentrations at pharmacological levels (Fig. 2b,c). Increased plasma levels of irisin improved recovery from EAE peak symptoms (Fig. 2d and Extended Data Fig. 2a,b). To assess the neuroprotective effects of irisin at a histological level, we analysed the number of NeuN^+^ neurons in the ventral horn and complete grey matter of the spinal cord, as well as the ChAT^+^ motor neurons in the ventral horn. Importantly, we detected a rescue of neuronal loss in all three analyses (Fig. 2e and Extended Data Fig. 2c).

The observed improvement in neuronal survival was accompanied by a reduction in the area covered by IBA1^+^ microglia and macrophages in the complete spinal cord (Fig. 2f) and in the non-lesion area with a numerical reduction in the lesion area (*P* = 0.0512) of irisin-treated mice (Extended Data Fig. 2d). There was no difference in astroglial reactivity, as indicated by glial fibrillary acidic protein (GFAP) expression, in either the numbers or cell size of GFAP^+^ cells (Fig. 2g and Extended Data Fig. 2e). Surprisingly, we did not detect differences in myelination, as indicated by luxol fast blue (LFB) staining or myelin basic protein labelling (Extended Data Fig. 2f,g). There were also no differences in the number, size or area of hematoxylin–eosin (H&E)^+^ inflammatory lesions (Extended Data Fig. 2h) or the number of infiltrating CD3^+^ T cells in the overall spinal cord and the lesion and non-lesion areas (Extended Data Fig. 2i).

As neuroinflammation and neurodegeneration also occur in the hippocampus during EAE (Extended Data Fig. 2j–l), we analysed the effects of irisin on hippocampal function using contextual fear conditioning (CFC) in the chronic phase of EAE. We confirmed that there was no false freezing detection related to disability of EAE mice (Extended Data Fig. 2m–o). In EAE, control mice lost their ability to discriminate contexts, while irisin treatment restored context discrimination (Fig. 2h). Furthermore, the loss of NeuN^+^ neurons in the hippocampus was reduced with irisin treatment (Fig. 2i). However, the area covered by IBA1^+^ microglia and macrophages as well as GFAP^+^ astrocytes was similar to controls (Extended Data Fig. 2p,q). To further assess the impact on overall neuroprotection of irisin in EAE, we analysed the number of BRN3A^+^ neurons (retinal ganglion cells (RGCs)) in the retina and detected an additional rescue of neuronal loss with irisin treatment (Fig. 2j), highlighting that irisin conveys broad neuronal resilience against neuroinflammation in several CNS compartments. Taken together, these results indicate that peripheral irisin can rescue neuronal loss and improve disease outcome in EAE.

## Peripheral irisin does not alter the peripheral and central immune response in EAE

**Fig. 3:**
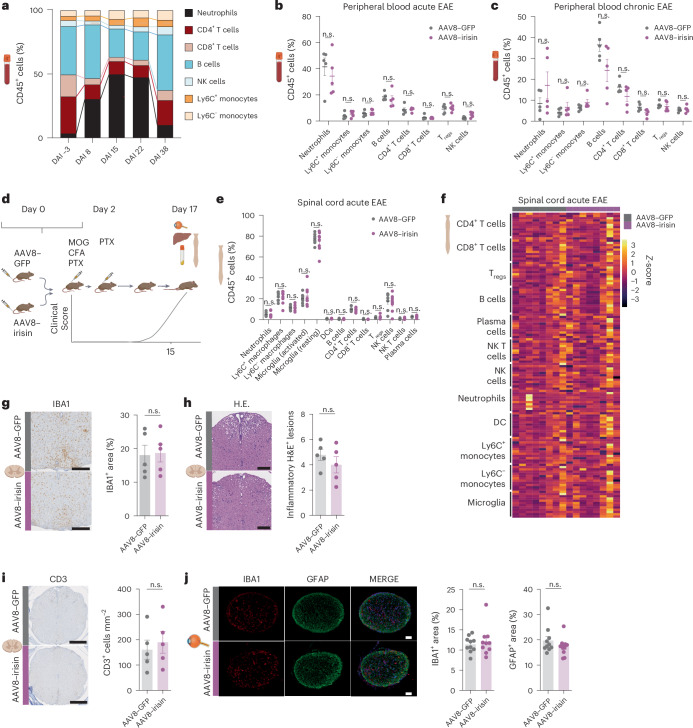
Peripheral irisin does not alter the peripheral and central immune response in EAE. **a**–**c**, WT mice were injected with AAV8–GFP or AAV8–irisin–Flag via the tail vein with subsequent EAE induction, longitudinal blood draws and tissue collection at the end of the experiment. The figure shows the relative quantification by flow cytometry of peripheral blood immunecell subsets during the course of EAE (mean of AAV8–GFP injected mice is shown, *n*/group = 5). DAI, day after immunization (**a**). Relative quantification by flow cytometry of indicated immune-cell subtypes at the peak of EAE (**b**) and the chronic phase of EAE (**c**) (AAV8–GFP *n* = 5, AAV8–irisin–Flag *n* = 5) is also shown. **d–j**, WT mice were injected with AAV8–GFP or AAV8–irisin–Flag via the tail vein with subsequent EAE induction, and the liver, plasma, spinal cord and optic nerve were collected at the peak of EAE. The figure shows the experimental layout (**d**) and the relative quantification by flow cytometry of indicated CNS-infiltrating or immune-cell subtypes (**e**) and their activation markers and cytokine expression (heat map with normalized mean values (*z*-score)) (**f**) in the spinal cord of EAE mice at peak of EAE (AAV8–GFP *n* = 8, AAV8–irisin–Flag *n* = 8). Representative images and analysis of histopathological stainings of IBA1^+^ microglia and CNS macrophages (**g**), H&E^+^ inflammatory lesion numbers (**h**) and infiltrating CD3^+^ T cells (**i**) in spinal cord sections (AAV8–GFP *n* = 5, AAV8–irisin–Flag *n* = 5) at peak of EAE are also shown, as well as representative images and analysis of immunofluorescence stainings of microglia and CNS macrophages (IBA1, AAV8–GFP *n* = 10 AAV8–irisin–Flag *n* = 10) and astrocytes (GFAP, AAV8–GFP *n* = 10 AAV8–irisin–Flag *n* = 10) in optic nerve sections of EAE mice at peak of EAE (**j**). Scale bar, 50 µm. Data are shown as mean ± s.e.m. of biological independent samples. Statistical analysis was performed with two-tailed unpaired *t*-tests followed by Bonferroni correction (**b**, **c**, **e** and **f**) and an unpaired two-tailed Student’s *t*-test (**g**–**j**). DCs, dendritic cells; NK, natural killer. Panel **d** created in BioRender; Rosenkranz, S. https://biorender.com/24elt4j (2025). Icons in **a**–**c**, **e**–**j** created in BioRender; Rosenkranz, S. https://biorender.com/b9z8gg0 (2025). Source data

**Extended Data Fig. 3:**
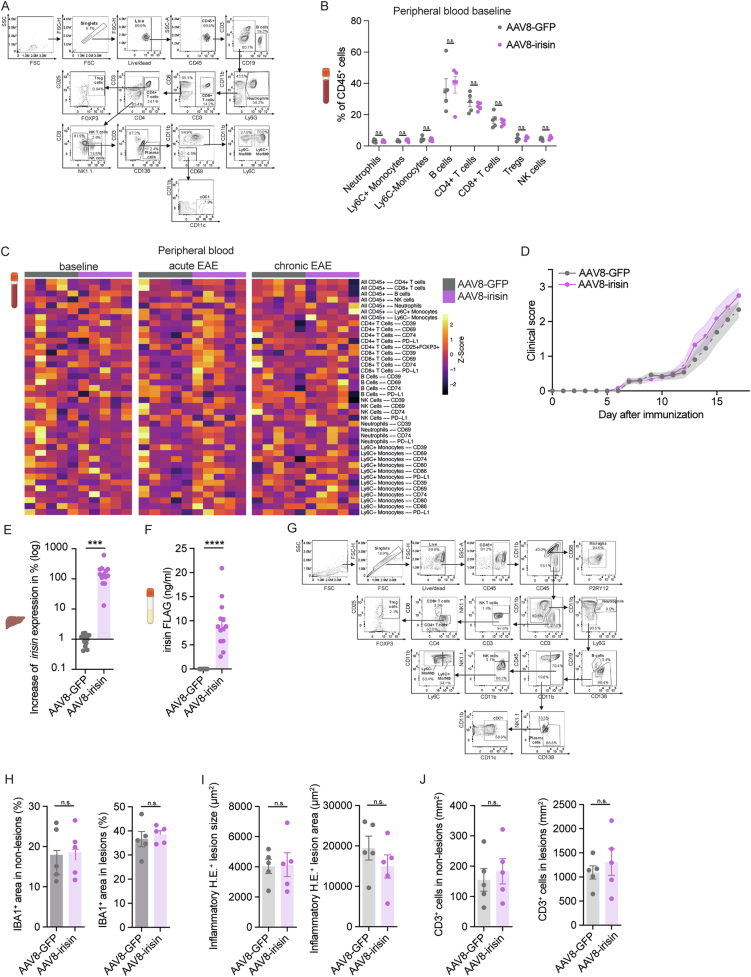
Peripheral irisin does not alter the peripheral and central immune response in EAE. **a**. Representative gating strategy for immunophenotyping of the peripheral blood by flow cytometry. **b, c**. WT were injected with AAV8-GFP or AAV8-irisin-FLAG via the tail vein with subsequent EAE induction and longitudinal blood draws. Relative quantification by flow cytometry of peripheral blood immune cell subsets at baseline (**b**) and their activation markers and cytokine expression (heatmap with normalized mean values (z-score)) at the indicated time points **(c)** (AAV8-GFP, n = 5, AAV8-irisin-FLAG, n = 5). **d-g**. WT mice were injected with AAV8-GFP or AAV8-irisin-FLAG via the tail vein with subsequent EAE induction. At peak of EAE, tissues were collected. Mean clinical scores of AAV8-GFP mice undergoing EAE for flow cytometry and histological analysis at peak of EAE (AAV8-GFP, n = 13, AAV8-irisin-FLAG, n = 13) **(d)**, qRT-PCR for ‘irisin’ mRNA expression in the liver normalized to the mean of GFP control *P* = 0.0003 (**e**), irisin plasma levels by ELISA *P* = < 0.0001 **(f)** (AAV8-GFP, n = 13, AAV8-irisin-FLAG, n = 13), representative gating strategy for CNS immunophenotyping of the spinal cord by flow cytometry **(g)**. **h**. Analysis of histopathological stainings of IBA1^+^ microglia and CNS macrophages in lesion and non-lesion areas of spinal cord sections (AAV8-GFP, n = 5, AAV8-irisin-FLAG, n = 5) at peak of EAE. **i**. H.E.^+^ inflammatory lesion size and area of spinal cord sections of AAV8-GFP (n = 5) and AAV8-irisin-FLAG (n = 5) mice at peak of EAE. **j**. Analysis of histopathological stainings of infiltrating CD3^+^ T-cells in lesion and non-lesion areas in spinal cord sections (AAV8-GFP, n = 5, AAV8-irisin-FLAG, n = 5) at peak of EAE. Data as mean ± SEM of biological independent samples. Statistical analysis was performed with two-tailed Mann–Whitney U test of the area under the curve (**d**), unpaired two-tailed Student’s t-test (**e, f, h-j**), and unpaired two-tailed Student’s t-test followed by Bonferroni correction **(b)**. ***p < 0.001, ****p < 0.0001. Icons in **b**, **c**, **e**, **f** created in BioRender; Rosenkranz, S. https://biorender.com/b9z8gg0 (2025). Source data

As EAE, similar to MS, presents a canonical inflammatory, autoimmune-driven pathology, we next examined whether the observed neuroprotective benefits may be related to direct immunomodulatory effects of peripherally elevated irisin levels. To examine the effect of peripheral delivered irisin on the peripheral inflammatory responses associated with EAE progression, we collected blood samples longitudinally from irisin-treated and control mice 3 days before immunization for EAE (baseline, day −3), at the onset of symptoms (day 8), in the acute phase (day 15), during the chronic phase (day 22) and at the end of EAE (day 38) (Fig. 3a). The relative proportions and activation status of major subsets of CD45^+^ immune cells were analysed by multiplex spectral flow cytometry (Extended Data Fig. 3a). EAE progression had a pronounced effect on peripheral immune composition, with the relative proportions of myeloid cells, particularly neutrophils, strongly increased, while the relative proportion of lymphocytes, including CD4^+^ and CD8^+^ T cells and B cells, decreased with the onset of EAE (Fig. 3a). By the end of the chronic phase, the relative percentages of CD45^+^ immune-cell subpopulations returned to baseline. No significant differences in the relative abundances of peripheral blood immune-cell subsets or their activation status were observed between the irisin-treated and control mice during the EAE course (Fig. 3b,c, Extended Data Fig. 3b,c and Supplementary Data 1).

To assess the effects of peripheral irisin on neuroinflammation and inflammatory infiltration into the CNS, tissues from irisin-treated and control mice were collected at the peak of disease (Fig. 3d and Extended Data Fig. 3d). We first confirmed *irisin* overexpression in the liver and elevated irisin levels in the plasma (Extended Data Fig. 3e,f). Detailed flow cytometry analysis of the spinal cord samples (Extended Data Fig. 3g) showed no significant changes between irisin-treated and control mice in the proportions of infiltrating immune cells, the relative percentages of activated CD45^+^ microglia or in the activation status of immune-cell subsets, as indicated by surface activation markers and cytokine production (Fig. 3e,f and Supplementary Data 2). Similarly, there were no significant differences between irisin-treated and GFP control animals in the area of IBA1^+^ microglia and macrophages in spinal cord lesion and non-lesion area (Fig. 3g and Extended Data Fig. 3h). We also found similar numbers of H&E^+^ inflammatory lesions, lesion size and lesion area (Fig. 3h and Extended Data Fig. 3i) and similar numbers of infiltrating CD3^+^ T cells in the lesion and non-lesion area (Fig. 3i and Extended Data Fig. 3j). Furthermore, no difference in the activation of IBA1^+^ microglia and GFAP^+^ astrocytes was found in the optic nerve at the peak of the disease (Fig. 3j). Taken together, these findings indicate that the observed functional neuroprotective effects of irisin are independent of inflammatory immune responses in EAE.

## Peripheral irisin induces a neuroprotective gene programme in EAE

**Fig. 4:**
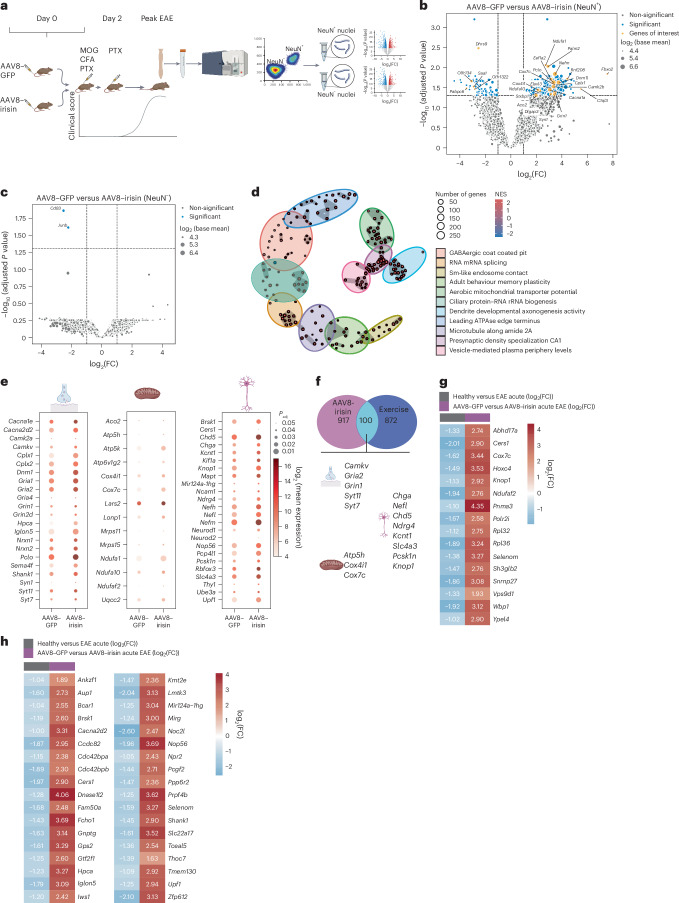
Peripheral irisin induces a neuroprotective gene programme in spinal cord neurons in EAE. **a**, WTs were injected with AAV8–GFP or AAV8–irisin–Flag via the tail vein with subsequent EAE induction. At the peak of EAE, neuronal and non-neuronal nuclei were isolated and sequenced. **b**,**c**, Volcano plot of gene expression analysis by DESeq2 in NeuN^+^ (**b**) and NeuN^−^ nuclei (**c**) of EAE mice at the peak of EAE (AAV8–GFP *n* = 3, AAV8–irisin–Flag *n* = 3). Significant genes are highlighted in blue. Genes of interest are highlighted in yellow. Applied significance thresholds (dashed lines): log_2_(FC) >|1| and FDR-adjusted (FDR-adj) *P* < 0.05. **d**, Enrichment map of regulated GO terms generated using GSEA, with each dot representing a distinct gene set. The edge thickness and length correlate with the similarity between GO terms. **e**, Dot plots showing synaptic, mitochondrial and neuronal DEGs of interest. In each, the hue and size of the dot represent the log_2_ (mean expression) and FDR-adj *P* value, respectively. **f**, Comparison of irisin treatment effects in spinal cord NeuN^+^ nuclei from EAE mice with reported running effects in spinal cord neurons from young and old mice (Sun et al.^33^). Selected overlapping DEGs are shown. **g**, Comparison of irisin treatment effects in spinal cord NeuN^+^ nuclei from EAE mice with reported acute EAE effects in spinal cord motor neurons by Bacterial Artificial Chromosome–Translating Ribosome Affinity Purification (BAC-TRAP; Schattling et al.^27^). DEGs present in both datasets are shown with their respective log_2_(FC). **h**, Comparison of irisin treatment effects in spinal cord NeuN^+^ nuclei from EAE mice with reported acute EAE effects in spinal cord NeuN^+^ nuclei from EAE and healthy control mice using RNA-seq (Woo et al.^29^). DEGs present in both datasets are shown with their respective log_2_(FC). Statistical analysis was performed with a two-sided Wald test followed by Bonferroni–Hochberg correction (**b**, **c** and **e**). Panel **a** created in BioRender; Rosenkranz, S. https://biorender.com/cq34wj3 (2025). Icons in **e**, **f** created in BioRender; Rosenkranz, S. https://biorender.com/b9z8gg0 (2025).

**Extended Data Fig. 4:**
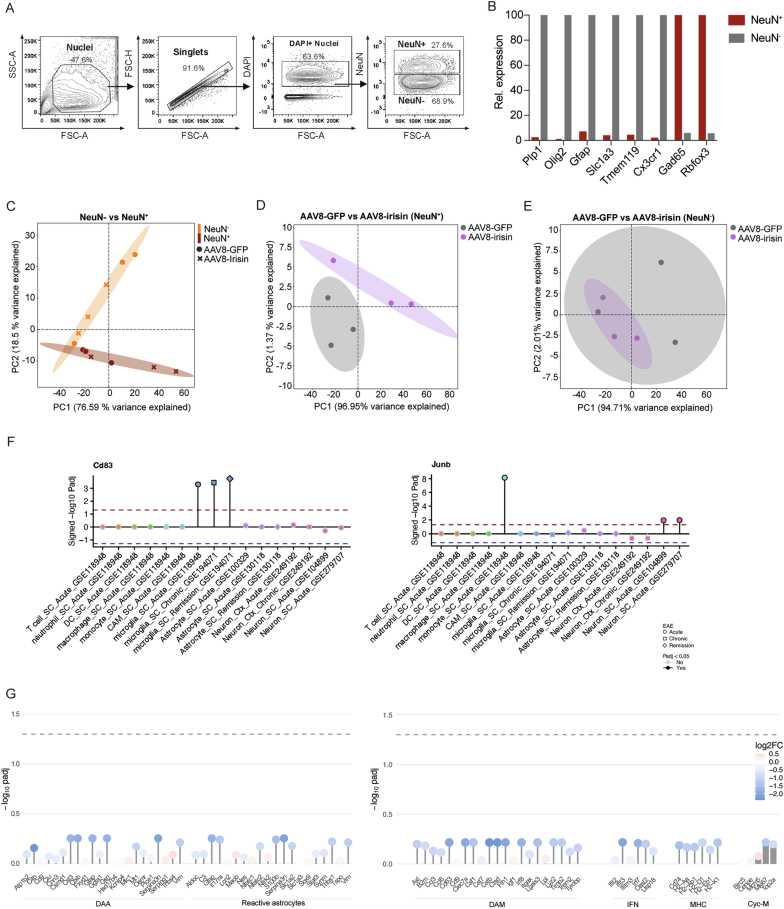
Peripheral irisin induces a neuroprotective gene program in spinal cord neurons in EAE. **a**. Representative gating strategy of neuronal nuclei from mouse spinal cord stained with DAPI and anti-NeuN. **b**. Relative qPCR mRNA expression of healthy spinal cord NeuN^+^ sorted nuclei and NeuN^−^ sorted nuclei of marker genes for oligodendrocytes (*Plp1, Olig2*), astrocytes (*Gfap, Slc1a3*), microglia (*Tmem119, Cx3cr1*) and neurons (*Gad65, Rbfox3*) in one wildtype mouse. **c-e**. WT were injected with AAV8-GFP or AAV8-irisin-FLAG via the tail vein with subsequent EAE induction. At peak of EAE, neuronal and non-neuronal nuclei were isolated and sequenced. Principal component analysis of NeuN^+^ and NeuN^−^ nuclei (**c**), NeuN^+^ nuclei of AAV8-GFP and AAV8-irisin-FLAG (**d**) and NeuN^−^ nuclei of AAV8-GFP and AAV8-irisin-FLAG (**e**). Each dot corresponds to one animal (AAV8-GFP, n = 3, AAV8-irisin-FLAG, n = 3). Ellipses indicate 95% confidence regions for each group. **f**. Analysis of the indicated published RNA-sequencing datasets of the spinal cord in different cell populations during EAE. **g**. Comparison of published^30–32^ activated astrocyte markers and activated microglia gene signatures with AAV8-GFP or AAV8-irisin-FLAG NeuN^−^ nuclei. The dashed lines represent an FDR-adjusted P-value < 0.05. Statistical analysis was performed with a two-sided FDR-corrected Wald test for GSE118948, an unpaired two-sided FDR-corrected Wilcoxon rank-sum test (**f-g**). Icons in **a**, **b** created in BioRender; Rosenkranz, S. https://biorender.com/b9z8gg0 (2025). Source data

To investigate the direct effects of peripherally elevated irisin levels on spinal cord neurons, we performed unbiased transcriptional profiling of NeuN^+^ and NeuN^−^ nuclei isolated from the spinal cord of irisin-treated and control mice at the peak of EAE (Fig. 4a and Extended Data Fig. 4a). We confirmed enrichment of neurons in the NeuN^+^ fraction by quantitative PCR (qPCR) using neuronal and non-neuronal marker genes and in our RNA sequencing (RNA-seq) data by principal component analysis (Extended Data Fig. 4b,c). To determine cell type-specific transcriptional responses by irisin in EAE, we performed differential gene expression analysis. Strikingly, in the NeuN^+^ neuronal nuclei, we found 1,017 differentially expressed genes (DEGs) with irisin treatment (Fig. 4b and Extended Data Fig. 4d). By contrast, only two genes were differentially regulated by irisin in the NeuN^−^ fractions, namely *Cd83* and *Junb*, which were both decreased in the irisin-treated mice (Fig. 4c and Extended Data Fig. 4e). *Cd83* and *Junb* are mainly expressed in immune cells, including microglia^22,23^. Analysis of published sequencing datasets of the spinal cord in different cell populations during EAE^24–29^ revealed that both genes were upregulated in monocyte-derived macrophages and microglia during EAE (Extended Data Fig. 4f). Using published classifications for disease-associated astrocytes^30^ and reactive astrocytes^31^ as well as for disease-associated microglia and other recently defined single-cell (sc)/single-nucleus (sn)RNA-seq-based microglial subtypes^32^, we did not observe differences between irisin-treated and GFP-treated mice in EAE in any astrocyte or microglial subtype (Extended Data Fig. 4g).

Gene set enrichment analysis (GSEA) of the NeuN^+^ nuclei identified multiple clusters of Gene Ontology (GO) terms indicating an upregulation of genes involved in aerobic mitochondrial transporter potential, presynaptic density and dendrite development (Fig. 4d). Irisin-induced DEGs in neurons included classical neuronal markers (for example, *Rbfox3*, *Thy1* and *Nefl*) as well as synaptic (for example, *Gria2*, *Syt7* and *Syt11*) and mitochondrial (for example, *Atp5h*, *Cox4i1* and *Ndufa1*) genes (genes of interest are highlighted in Fig. 4e).

Next, we compared the transcriptional response induced by irisin treatment in EAE to the transcriptional response induced by running in spinal cord neurons as previously published^33^. We found that approximately 10% of the transcriptional responses overlapped, meaning that 100 genes, including many neuronal, synaptic and mitochondrial genes, were upregulated in spinal cord neurons by both (genes of interest are highlighted in Fig. 4f) (Supplementary Data 3) We additionally compared our dataset to two published datasets of genes dysregulated in acute EAE in spinal cord motor neurons^27,29^. We found that 50 coding genes, which are dysregulated by EAE, were rescued by irisin delivery in EAE (Fig. 4g,h and Supplementary Data 4 and 5). Among these 50 genes, 4 genes were related to synapses (*Shank1*, *Hpca*, *Cacna2d2* and *Iglon5*) and 2 genes (*Ndufaf2* and *Cox7c*) were related to mitochondria, including *Cox7c*, which is part of complex IV, indicating potential counteractive mechanisms of irisin.

## Peripheral irisin and exercise rescue synapse loss and mitochondrial activity in EAE

**Fig. 5:**
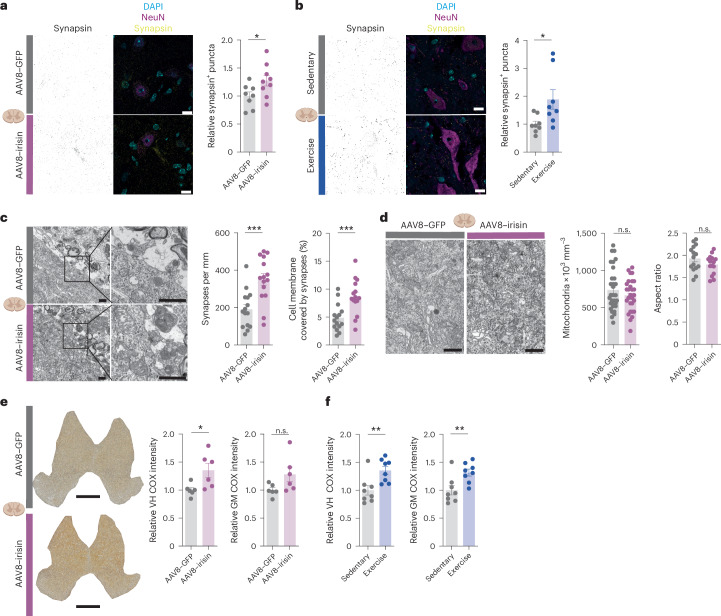
Irisin induces rescue of synaptic loss and enhanced ventral horn mitochondrial activity in EAE. **a**,**b**, Representative images and analysis of immunofluorescence stainings of synapses (synapsin 1/2) and neurons (NeuN) in spinal cord ventral horn (VH) of AAV8–GFP (*n* = 8) and AAV8–irisin–Flag (*n* = 9) (*P* = 0.0418) (**a**) and WT exercise (*n* = 8) and WT sedentary (*n* = 8) (*P* = 0.0292) mice (**b**) in the chronic phase of EAE. Scale bars, 10 µm. **c**, Representative images and analysis of electron microscopic images of the number of synapses (*P* = 0.0005) and percentage of cell membrane covered with synapses per motor neuron (*P* = 0.001) of AAV8–GFP (*n* = 15) and AAV8–irisin–Flag (*n* = 15) mice at the peak of EAE (five motor neurons per animal, three animals per group). Scale bars, 1 µm. **d**, Representative images and analysis of electron microscopic images of the numbers of mitochondria per motor neuron (AAV8–GFP (*n* = 31) and AAV8–irisin–Flag (*n* = 28); 9–11 motor neurons per animal, 3 animals per group) and aspect ratio of mitochondria per motor neuron (AAV8–GFP *n* = 15, AAV8–irisin–Flag *n* = 15; five motor neurons per animal, three animals per group) at the peak of EAE. Scale bar, 1 µm. **e**,**f**, Representative images and quantification of COX histochemistry of spinal cord sections of AAV8–GFP (*n* = 6) and AAV8–irisin–Flag (*n* = 6) mice VH (*P* = 0.0248) and grey matter (GM; *P* = 0.0704) (**e**) and WT exercise (*n* = 8) and WT sedentary (*n* = 8) mice VH (*P* = 0.007) and GM (*P* = 0.0093) (**f**) in the chronic phase of EAE. Scale bar, 400 µm. Data are shown as mean ± s.e.m. of biological independent samples. Statistical analysis was performed using an unpaired two-tailed Student’s *t*-test (**a**–**f**). *P < 0.05, **P < 0.01, ***P < 0.001. Icons in **a**–**e** created in BioRender; Rosenkranz, S. https://biorender.com/b9z8gg0 (2025). Source data

**Extended Data Fig. 5:**
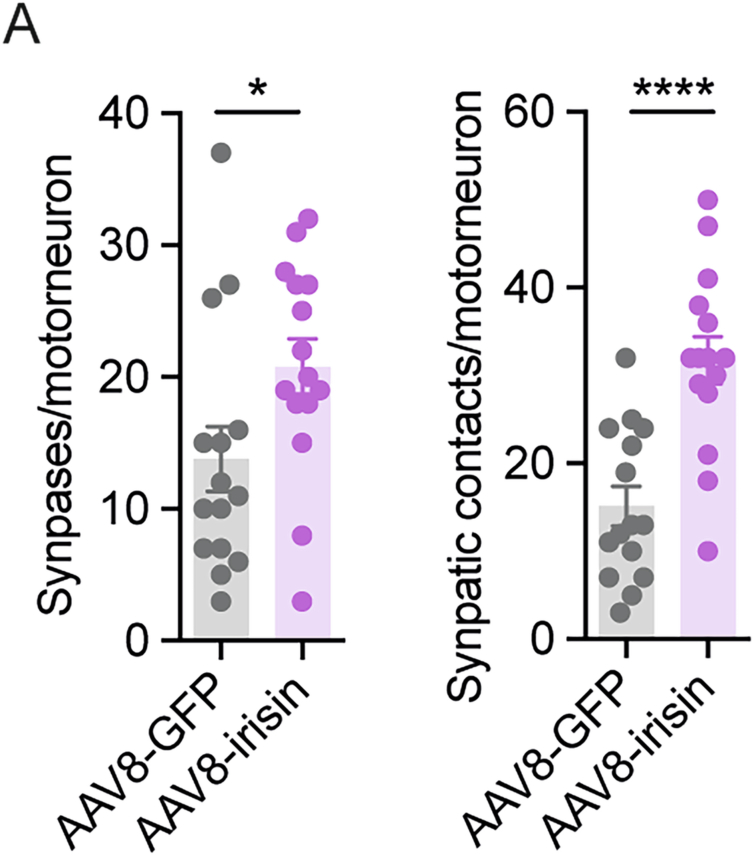
Irisin induces rescue of synaptic loss and enhanced ventral horn mitochondrial activity in EAE. **a**. Analysis of electron microscopic images of the absolute number of synapses *P* = 0.0381 and synaptic contacts per motorneuron *P* < 0.0001 of AAV8-GFP (n = 15) and AAV8-irisin-FLAG (n = 15) mice at peak of EAE (5 motorneurons per animal, 3 animals per group). Data as mean ± SEM of biological independent samples. Statistical analysis was performed with unpaired two-tailed Student’s t-test (**a**). *p < 0.05, ****p < 0.0001. Source data

To further validate the mechanistic impact of our findings, we analysed the density of synapses between the irisin-treated and control mice using immunofluorescence and confocal microscopy. Indeed, we detected a rescue of synaptic loss in the ventral horn during EAE by irisin (Fig. 5a) in the chronic phase. Similarly, exercise in EAE mice rescued synaptic loss compared with sedentary animals (Fig. 5b) in the chronic phase. Using electron microscopy, we confirmed rescue of the number of synapses, synaptic contacts and percentage of cell membrane covered by synapses on the ventral horn motor neurons at peak of disease (Fig. 5c and Extended Data Fig. 5a). Mitochondrial number, area and elongation were unchanged at the peak of disease (Fig. 5d). However, given that downregulation of electron transport chain transcripts in EAE compromises oxidative phosphorylation^34^, we tested whether irisin restores mitochondrial function. Indeed, complex IV activity was increased in the ventral horn of irisin-treated mice (Fig. 5e) and in running animals in the chronic phase (Fig. 5f), indicating enhanced oxidative phosphorylation.

## Irisin binds to spinal cord motor neurons in EAE

**Fig. 6:**
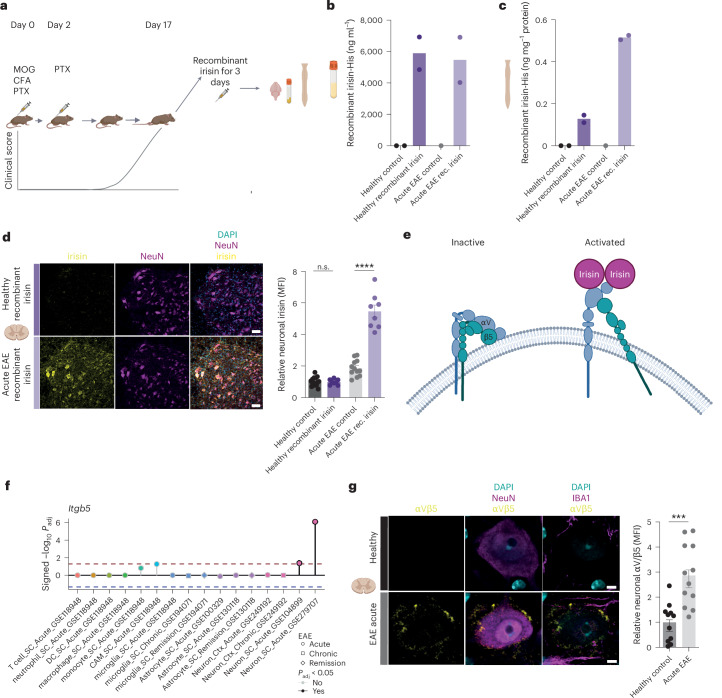
Irisin binds to neurons in the spinal cord in EAE. **a**, WT healthy or WT EAE mice at the peak of EAE were injected with recombinant irisin and plasma, and the brain and spinal cord were analysed. **b**, Irisin plasma levels measured by ELISA (healthy PBS *n* = 2, healthy recombinant irisin *n* = 2, EAE PBS *n* = 1, EAE recombinant irisin *n* = 2) in WT mice. **c**, Irisin spinal cord levels measured by ELISA (healthy PBS *n* = 2, healthy recombinant irisin *n* = 2, EAE PBS *n* = 1, EAE recombinant irisin *n* = 2) in WT mice. **d**, Representative images and analysis of irisin MFI in NeuN^+^/DAPI^+^ neurons in healthy and EAE animals receiving recombinant irisin and respective controls: *P* = < 0.0001 (healthy *n* = 16, healthy recombinant irisin *n* = 8, EAE *n* = 12, EAE recombinant irisin *n* = 8 (four neurons per animal)). Scale bar, 50 µm **e**, Schematic illustration of an integrin αV/β5 receptor. **f**, Analysis of the indicated published sequencing datasets in different cell populations during EAE showing significant upregulation of *Itgb5* expression exclusively in spinal cord neurons. The dashed lines represent an FDR-adjusted *P* value <0.05. **g**, αV/β5 MFI in motor neurons of healthy (*n* = 11) and of EAE mice (*n* = 12) *P* = 0.0003 at the peak of EAE (two to three motor neurons per animal), Scale bar, 10 µm. Data shown as mean ± s.e.m. of biological independent samples. Statistical analysis was performed using a two-way ANOVA with Šídák’s multiple comparisons test (irisin × EAE *P* < 0.0001, irisin *P* < 0.0001, EAE *P* < 0.0001) (**d**), unpaired two-tailed Student’s *t*-test (**g**) and two-sided FDR-corrected Wald test for GSE118948 and unpaired two-sided FDR-corrected Wilcoxon rank-sum test (**f**). ***P < 0.001, ****P < 0.0001. n.s., not significant. Panels **a** and **e** created in BioRender: **a**, Rosenkranz, S. https://biorender.com/fr6lo5r (2025); **e**, Rosenkranz, S. https://biorender.com/l83n6ne (2025). Icons in **b**–**d**, **g** created in BioRender; Rosenkranz, S. https://biorender.com/b9z8gg0 (2025). Source data

**Extended Data Fig. 6:**
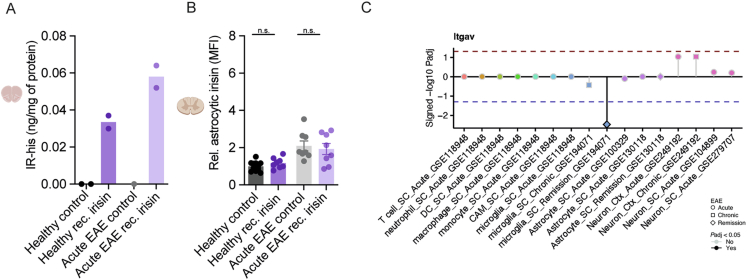
Irisin binds to spinal cord neurons in EAE. **a**. Irisin prefrontal cortex levels measured by ELISA (Healthy PBS n = 2, Healthy recombinant irisin n = 2, EAE PBS n = 1, EAE recombinant irisin n = 2) in WT mice. **b**. Irisin mean fluorescence intensity in GFAP^+^/DAPI^+^ - astrocytes in healthy (n = 11), healthy recombinant irisin (n = 8), EAE (n = 8), EAE recombinant irisin (n = 8) animals (1–4 astrocytes per animal). **c**. Analysis of the indicated published sequencing datasets in different cell populations during EAE, showing no regulation of *Itgav* expression in neurons during EAE but downregulation in microglia. The dashed lines represent an FDR-adjusted p-value < 0.05. Data as mean ± SEM of biological independent samples. Statistical analysis was performed with Two-Way ANOVA with Šídák’s multiple comparisons test (irisin x EAE: n.s., irisin: n.s., EAE: p < 0.0001), n.s. = not significant (**b**) and two-sided FDR-corrected Wald test for GSE118948 an unpaired two-sided FDR-corrected Wilcoxon rank-sum test (**c**). Source data

Next, we examined whether direct binding of irisin may take place in the CNS to mediate the observed beneficial effects. As endogenous irisin is not detectable by available antibodies^9^, we injected recombinant irisin or PBS as vehicle control for three consecutive days at peak of EAE and in healthy control WT mice (Fig. 6a). We detected similar plasma irisin levels in healthy and EAE animals receiving recombinant irisin (Fig. 6b). However, recombinant irisin levels trended higher in the spinal cord and the prefrontal cortex in EAE animals (Fig. 6c and Extended Data Fig. 6a). Importantly, we detected higher levels of irisin protein specifically in NeuN^+^ neurons in the ventral horn in EAE animals receiving recombinant irisin in comparison to healthy animals injected with recombinant irisin (Fig. 6d). By contrast, no such binding was observed on GFAP^+^ astrocytes (Extended Data Fig. 6b). We observed an increased irisin signal in EAE compared with healthy mice, either caused by unspecific staining or increased endogenous irisin binding. However, the irisin signal in neurons was still significantly increased approximately threefold in irisin-injected EAE mice over non-irisin-injected control EAE mice but not in astrocytes (Fig. 6d and Extended Data Fig. 6b). Taken together, these data suggest increased binding of irisin on spinal cord neurons in EAE.

Previous work has proposed that irisin binds to the αVβ5 integrin receptor^5,35^, which consists of an αV and a β5 subunit^34^ (Fig. 6e). Whereas *Itgav*, the gene encoding for the αV subunit, is expressed in healthy neurons, *Itgb5*, the gene encoding for the β5 subunit, is not, thereby preventing irisin binding to neurons via αV/β5 under physiological conditions^36^. Of note, analysis of different RNA-seq datasets^24–29^ revealed an upregulation of *Itgb5* specifically in spinal cord motor neurons in EAE (Fig. 6f), whereas no difference was detected for *Itgav* (Extended Data Fig. 6c*)*. We confirmed upregulation of the αVβ5 integrin receptors on the neuronal surface of ventral horn NeuN^+^ neurons in acute EAE by immunofluorescence (Fig. 6g), which could explain the increased recombinant irisin levels in neurons in EAE. Taken together, our data point towards a direct neuroprotective mechanism of irisin specifically enabled in inflammatory neurodegeneration.

## Discussion

Capturing the molecular mediators of the neuroprotective effects of exercise holds great promise for the development of disease-modifying treatments for MS. Here, we demonstrate a direct link between irisin, a bona fide exercise-induced molecular mediator, and structural and functional neuroprotection in the animal model of MS. We show that peripherally delivered irisin confers neuroprotection in EAE. While the neuroprotective effects of irisin have been reported previously in mouse and cellular models of Alzheimer’s disease and Parkinson’s disease, the observed effects in a multiple sclerosis mouse model are noteworthy because of the autoimmune neuroinflammatory nature of this disease^3,4^.

Intriguingly, we found that the protective effects of irisin overexpression are not associated with substantial changes in the relative proportion or functional phenotype of circulating or CNS-infiltrating immune cells at the inflammatory peak of EAE progression. At chronic stages, we found lower numbers of IBA1^+^-activated microglia in the spinal cord of irisin-treated animals, which probably correlates with reduced neuronal cell death and a consequent reduction in damage-associated molecular patterns (DAMPs)^37^. Furthermore, we have not observed direct effects on the activation or regulatory response of astrocytes in EAE, even though earlier reports in Alzheimer’s disease found that irisin can modulate astrocyte functions^3,5^. A recent study suggested protective effects in EAE by extremely low-dose recombinant irisin, which would have been eliminated from plasma in less than 1 h (ref. ^38^). Interestingly, this study ascribed the protective effects to reduced microglia activation and did not investigate direct effects on neurons.

Strikingly, we find that the neuroprotective effects of irisin appear to be directly mediated by increased neuronal survival, ventral horn synaptic preservation and enhanced neuronal mitochondrial activity, as shown by immunofluorescence, immunohistochemistry, RNA-seq and electron microscopy. Mitochondrial deficits are one of the main drivers of neurodegeneration in MS, and increasing mitochondrial function rescues inflammatory neurodegeneration in EAE^34,39,40^. Interestingly, the observed modulation of mitochondria aligns with the original role described for irisin^8^. Similarly, synaptic loss is a primary cause for observed neurobehavioural deficits in MS and EAE^41–43^. Our observed rescue of ventral horn synapses by running and peripheral irisin is consistent with previous reports showing a synaptic rescue by exercise in the hippocampus during EAE^44^. Lastly, running exercise failed to confer neuroprotection in *Fndc5*-KO mice. These results highlight a specific, exercise-mediated neuroprotective pathway involving irisin in MS.

Importantly, we provide evidence that irisin exerts direct neuroprotective effects by binding to motor neurons in the ventral horn in EAE animals at peak disease, coinciding with upregulation of its proposed receptor. Further investigation will be required to clarify the dynamics of this newly observed protective mechanism, which appears to upregulate irisin receptors on the surface of damaged motor neurons in the spinal cord.

Together, our findings strengthen the argument that irisin may be an important molecular mediator of neuroprotection in this context. As the effects of exercise are complex and pleiotropic, it is unlikely that all the observed benefits of exercise in MS are mediated by a single molecule. Of note, the EAE running experiments in WT and *Fndc5*-KO mice were not performed at the same time, and the *Fndc5*-KO mice were slightly older at the beginning of the experiment. However, an approximate 2-week age difference at EAE immunization at that age was not statistically significant or biologically relevant for the EAE disease model.

In summary, our results from using irisin in a therapeutic regimen strongly support irisin as an attractive drug target to counteract neurodegeneration in the progressive phase of MS, pending validation in future studies.

## Methods

### Animal procedures and ethics statement

All animal procedures were approved by the Institutional Animal Care and Use Committee (IACUC) of the Massachusetts General Hospital (MGH) or the State Authority of Hamburg, Germany. C57BL/6J (000664, JAX) were used as WT mice. We published the generation and phenotyping of the global *Fndc5*-KO mice in Kim et al.^35^ and Islam et al.^3^. *Fndc5*-KO mice were maintained with het-to-het breedings on a C57BL6 background. All experimental animals were housed in the specific pathogen-free environment animal facility at MGH with a regular 12 h light and 12 h dark cycle from 07:00 to 19:00, at 20–22 °C and 30–70% humidity. All procedures were carried out during the light-phase. Mice had free access to water and standard chow (Prolab IsoPro RMH 3000, or Altromin 1310, irradiated). Mice were group-housed except for running experiments. For the WT voluntary free-wheel running EAE experiments, 6–8-week-old male mice were used. For all other experiments, 7–13-week-old female mice were used. Assignment to experimental groups was random. Tissues were collected at the indicated timepoints. All procedures were carried out in accordance with the Animal Research: Reporting of in Vivo Experiments (ARRIVE) guidelines^45^. Genotype for *Fndc5*-KO mice was confirmed by PCR from tail snips.

### EAE

We immunized mice subcutaneously with either 200 μg MOG_35–55_ peptide (Schafer-N) in Complete Freund’s Adjuvants (CFA) (Difco, cat. no. DF0639–60–6) containing 2 mg ml^−1^ *Mycobacterium tuberculosis* (Difco, cat. no. DF3114-33-8) or with the commercially available kit (Hooke Kit MOG_35–55_/CFA). In addition, we injected 100 ng (WT voluntary wheel-running EAE experiment) or 200 ng pertussis toxin (all other experiments) (Calbiochem, cat. no. CAS70323-44-3, or Emulsion PTX, cat. no. EK-2110) intraperitoneally (i.p.) on the day of immunization and 48 h later. We scored animals daily for clinical signs by the following system: 0, no clinical deficits; 1, tail weakness; 2, hind limb paresis; 3, partial hind limb paralysis; 3.5, full hind limb paralysis; 4, full hind limb paralysis and fore limb paresis; 5, premorbid or dead. Animals reaching a clinical score ≥4 were killed according to the regulations of the local animal welfare legal authorities. All animals were monitored for dehydration and weighed daily. DietGel 76A gels (Clear H2O) were provided to support nutrition. The investigators were blind to the treatment in the AAV8–irisin or recombinant irisin cohorts. Behavioural experiments and tissue collections were performed at the indicated timepoints.

### Tail-vein injections of AAV8s

Mice were injected into the tail vein with AAV8–GFP or AAV8–irisin–Flag (1 × 10^10^ GC per mouse; UPenn Vector Core) diluted in PBS to a final volume of 100 μl. For details of the generation of the AAV vector, see Islam et al. and Kim et al.^3,35^. EAE was induced on the same day as the AAV injections.

### Recombinant irisin–His injections

Recombinant human/mouse irisin containing a C-terminal His tag was produced in HEK293 (Proteos). The endotoxin level was determined to be <0.500 EU ml^−1^. The final irisin–His has the correct predicted molecular weight of ~15 kDa in the unglycosylated state and ~25 kDa in the glycosylated form. Healthy WT mice or mice at the peak of EAE were injected with 5 mg kg^−1^ body weight sterile recombinant irisin–His or PBS i.p. for 2 days, followed by a third injection at 10 mg kg^−1^ body weight on the next day. After 1 h of the last injection, the animals were killed and tissues collected as described below.

### Voluntary free-wheel running paradigm

Mice were weighed and placed individually in type III cages with free access to functional running wheels (Ø, 13 cm), which were either blocked with a magnet (sedentary) or not blocked (voluntary free-wheel running). No other enrichment was present. Running activity was tracked using a magnetic contact-counting instrument (set-up built by Torsten Renz). Similarly, in the *Fndc5*-KO voluntary wheel-running cohorts, animals were placed in individual cages with either blocked (sedentary) or not-blocked (voluntary wheel running) stainless-steel running wheels (Starr Life Sciences), and running activity was tracked every hour using a revolution counter (VitalView Animal Activity v1.4, Starr Life Science). Running distance and body weights were monitored a minimum of twice per week. After 8 weeks of voluntary access to running wheels or blocked running wheels, EAE was induced. Mice that did not run a minimum of 1 km per 24 h on average were excluded (one WT voluntary wheel-running EAE mouse excluded). We report all information on the basis of the recently published recommendations for minimal reporting criteria for MS exercise studies in animals^15^.

### CFC

CFC was used on day 35 and day 36 of EAE or in healthy age-matched WT mice. Mice were placed in a conditioning chamber (17 cm × 17 cm × 25 cm, Maze Engineers) with Plexiglas sides and a steel bar floor. They explored the chamber for 2 min before receiving two 2-s foot shocks (0.5 mA), spaced 2 min apart. Then, 1 min after the final shock, the mice were removed. A total of 24 h later, they were returned to the conditioning chamber (context A) for 3 min without receiving any additional shocks. Freezing behaviour during this session was recorded and analysed using ANY-maze software (Stoelting). Freezing served as a proxy for memory performance, as it reflected the mice’s recall of the previous shock experience in context A on day 1, expected to result in marked freezing episodes on day 2. After testing in context A, mice were placed in an alternate context chamber (context B) with no grid floor and newly patterned walls for 3 min, as before. Mice that did not exhibit freezing on day 1 after receiving shocks were excluded from analysis, as freezing could not be used as a measure of learning or memory for these animals. All tests were performed at the same time during the light phase in a dedicated behavioural suite. Experimenters were blinded to the treatment.

### Perfusion and tissue collection

Brain, spinal cord, liver, gastrocnemius muscle, epididymal/perimetrial fat pads, blood and eyeballs were collected for molecular, histological and ultrastructural analysis^34^. In the WT and the WT voluntary wheel-running experiment, mice were anesthetized i.p. with 100 µl solution (10 mg ml^−1^ esketamine hydrochloride (Pfizer) and 1.6 mg ml^−1^ xylazine hydrochloride (Bayer) dissolved in water) per 10 g of body weight. The epididymal fat pads were dissected and weighed. Then, mice were perfused with ice-cold PBS and the gastrocnemius muscle was dissected. After EAE, mice were perfused with 4% paraformaldehyde (PFA)^34^ and the remaining tissues were collected. For the WT cohort (CFC test), AAV8–irisin cohort, *Fndc5*-KO voluntary wheel running and recombinant irisin–His cohort, the mice were anesthetized with isoflurane and blood was collected via the vena cava. Then, mice were perfused with ice-cold PBS alone, ice-cold PBS followed by 4% PFA or ice-cold PBS followed by 4% PFA, 1% glutaraldehyde (GA; G6403, Sigma) in 0.1 M phosphate buffer (BB-185, Boston BioProducts) (for transmission electron microscopy (TEM)). As indicated below, tissues were either snap-frozen with liquid nitrogen, directly frozen in embedding solution (Tissue-Tek O.C.T. compound), fixed in 4% PFA or fixed in a mixture of 4% PFA, 1% GA in 0.1 M phosphate buffer. Gastrocnemius muscle samples were snap-frozen with liquid nitrogen and then frozen in embedding solution for cryosectioning.

### Irisin–Flag and irisin–His ELISA

At the end of the EAE experiments, where indicated, blood samples were collected in heparin-coated tubes (365985, BD Microtainer) and centrifuged to separate the plasma. The plasma fraction was stored at −80 °C until analysis. When indicated, prefrontal cortex and spinal cord tissue previously snap-frozen in liquid nitrogen were homogenized in RIPA buffer (R0278, Sigma) supplemented with protease (1861279, ThermoFisher Scientific) and phosphatase inhibitors (1862495, ThermoFisher Scientific). After protein quantification using a Pierce BCA protein assay kit (23225, ThermoFisher Scientific), the tissue supernatants normalized to the same total protein amount for all animals were used for analysis. For the irisin–Flag enzyme-linked immunosorbent assay (ELISA) assay, 96-well plates were coated with an anti-irisin capture antibody (MAB8880, R&D) in PBS and incubated overnight at 4 °C. The following day, plates were washed four times with 0.1% PBS with Tween (PBST), then blocked with 1% BSA for 1 h at room temperature (RT). After blocking, the plates were washed again with 0.1% PBST, and standards (0–200 ng ml^−1^) along with plasma samples were added to the wells, followed by a 2-h incubation at RT. Plates were then washed four times with 0.1% PBST and incubated with an anti-Flag detection antibody (14793S, CST) for 2 h at RT. After another wash with PBST, a horseradish peroxidase-conjugated secondary antibody was added for 30 min. Following additional washes, 3,3′,5,5′-tetramethylbenzidine (TMB) chromogen (ab171522, Abcam) was added as the detection reagent. Absorbance at 450 nm was measured using a plate reader (FLUOstar Omega, BMG Labtech) after stopping the reaction with stop solution (ab171529, Abcam). The irisin–His ELISA assay was performed similarly as above with the following adaptations: standards and samples were plated in a His Tag Antibody Plate (L00440C, GenScript) and incubated overnight at 4 °C, and an anti-irisin antibody (AG-25B-0027-C100, Adipogen) was used as detection antibody for 1 h at RT. The irisin concentration was determined by referencing a standard curve generated with either a recombinant irisin–Flag (AG-40B-0136-C010, Adipogen) or a recombinant irisin–His.

### Longitudinal blood collection

For longitudinal tracking of immune-cell subsets, blood was collected by submandibular venipuncture 3 days before immunization (day −3), at symptom onset (day 8), at symptom peak (day 15) and in the chronic phase of EAE (day 22). Blood was collected in heparinized tubes and cryoprotected using 55-5 BloodStor media (STEMCELL Technologies) following manufacturer’s instructions. Cryoprotected blood samples were stored in liquid nitrogen until use.

### Immunophenotyping by flow cytometry of blood and spinal cord tissue

To avoid technical batch effects, all blood samples collected during the longitudinal study were processed for flow cytometry at the same time, using the same reagents. Cryopreserved blood samples were washed and resuspended in PBS and stained using a Zombie NIR fixable dye (Biolegend) for 30 min. The cells were then washed and resuspended in PBS containing 1% fetal bovine serum, 0.01% sodium azide (RICCA Chemical) and 5% FcR blocking reagent (Miltenyi Biotec) for 10 min. Blocked cells were incubated for 30 min with fluorophore-conjugated primary surface antibodies (Supplementary Table 3). Surface-stained cells were washed and resuspended in fixation buffer (Biolegend) for 20 min, followed by permeabilization wash buffer (1×) (Biolegend). Permeabilized cells were incubated for 30 min with intracellular antibodies (Supplementary Table 3). All incubation steps were performed at 4 °C, protected from light. Cells were analysed on an Aurora spectral flow cytometer (Cytek) equipped with 355-nm, 405-nm, 488-nm, 561-nm and 640-nm lasers, using SpectroFlo software, version 3.3.0. At least 100,000 events were collected from each sample for analysis. Data were analysed using FlowJo software, version 10.8.1 (TreeStar).

### Spinal cord immunophenotyping

The animals received an intravenous injection of 250 μg brefeldin A, a macrolide that disrupts protein exocytosis, to allow better detection of cytokines by flow cytometry^46^. In total, 4 h after brefeldin injection, the mice were anesthetized and the spinal cords were rapidly extracted in ice-cold PBS, transferred to microfuge tubes for mechanical trituration and then enzymatically dissociated for 25 min at 37 °C, using a papain-based method according to the manufacturer’s specifications (Miltenyi Biotec). Before immunolabelling, myelin was removed from cell suspensions using commercially available myelin depletion beads (Miltenyi Biotec). Resulting cell suspensions were labelled for flow cytometry as described above, with the antibodies presented in Supplementary Table 4. Samples were analysed by spectral flow cytometry as described above, and at least 1,000,000 events were collected from each sample for analysis.

### Immunohistopathology

Cervical spinal cords were postfixed in 4% PFA for 60 min. Then, the tissue was dehydrated and cryoprotected in 30% sucrose solution in PBS for at least 1–2 days at 4 °C, frozen in embedding solution (Tissue-Tek O.C.T. compound) and cut into 12-μm-thick transverse cryosections. Brains or spines were postfixed for 24 h at 4 °C. Spines were decalcified, dehydrated and paraffinized and brains were dehydrated and paraffinized and cut into ~5-μm-thick transverse paraffin sections. Succinate dehydrogenase histochemistry (SDH)/cytochrome c oxidase (COX) histochemistry was performed according to the standard procedures of the University Medical Center Hamburg–Eppendorf (UKE) Mouse Pathology Facility. Briefly, gastrocnemius or cervical spinal cord sections were incubated for 60 min at 37 °C with SDH (0.2 M sodium succinate, 50 mM Tris-Cl and 50 mM MgCl_2_) or COX reaction media (diaminobenzidine tetrahydrochloride, cytochrome c and bovine catalase (all from Sigma) in 0.2 M phosphate buffer) and embedded with Aquatex (Merck). Images of tissue sections at 200× magnification were scanned using a Zeiss MIRAX MIDI Slide Scanner (Carl Zeiss, MicroImaging GmbH). Paraffin sections were stained for H&E, LFB and with antibodies directed against CD3, GFAP, NeuN, Chat, MBP and IBA1 that were visualized using the avidin–biotin complex technique with 3,3′-diaminobenzidine (brown stain) (UltraView Universal DAB Detection Kit, Roche) with the Ventana BenchmarXT (Roche) according to the standard procedures of the UKE Mouse Pathology Facility^34^. Images were analysed with QuPath version 0.3.0 software (https://qupath.github.io). Muscle fibres that were positive for SDH and COX were counted, and the percentage of positive-to-negative fibres or area of positive SDH staining was calculated. For quantification of complex IV activity in the spinal cord, for each annotated region (grey matter or ventral horn), the mean DAB optical density was calculated. Numbers and areas of H&E^+^ inflammatory foci per spinal cord section and numbers of Chat^+^ motor neurons per area were quantified manually. We used customized counting masks for CD3^+^ cells and NeuN^+^ cells and thresholding masks for the IBA1^+^ area, the GFAP^+^ area and the LFB- and MBP-stained area. All analysis conditions were standardized. For quantification, the hippocampus was divided into up to three sections per animal, the spinal cord was divided into up to four sections per animal and the mean per animal was calculated, which was used for subsequent statistical comparisons.

### Immunofluorescence

Brains or cervical spinal cords were postfixed in 4% PFA for 60 min. Then, the spinal cords were dehydrated and cryoprotected in 30% sucrose solution in PBS for at least 1–2 days at 4 °C, frozen in embedding solution (Tissue-Tek O.C.T. compound) and cut into 12-μm or 40-µm-thick transverse cryosections. Then, immunofluorescence staining was carried out on slides or free-floating sections^33^. Slices were washed three times with PBS, permeabilized with 0.25% Triton X-100 in 1× PBS for 15 min, blocked with either 10% normal donkey serum in PBS containing 0.05% Triton X-100 or 3% BSA, 3% normal goat serum and 0.1% Triton X-100 in 1× PBS for 45 or 60 min (followed by Fc block with fab fragment for 60 min at RT for the integrin αV/β5 staining) and subsequently stained overnight in a humidified slide staining system at 4 °C with primary antibodies (NeuN, Synapsin1/2, IBA1, irisin, integrin αV/β5; Supplementary Table 1). All additional steps were carried out at RT. Secondary antibodies were used according to Supplementary Table 2 for a 2-h incubation in a humidified chamber, and slices were embedded in ROTIMount FluorCare 4′,6-diamidino-2-phenylindole (DAPI) (Carl Roth, cat. no. HP20.1) or Fluoromount-G (E3325-RH35, SouthernBiotech). Paraffin-embedded spinal cord slices were dewaxed, rehydrated and processed by antigen retrieval for 10 min in boiling 10 mM sodium citrate buffer (pH 6.0). Slices were permeabilized with 0.1% Triton X-100 and blocked with 5% BSA in PBS with 0.1% Triton X-100 for 1 h, followed by incubation of the primary antibodies in 3% BSA/PBS with 0.1% Triton X-100 overnight at 4 °C. Images were captured in *Z*-stack mode using a confocal microscope with a 20× or 60×/0.8 objective (LSM 900, Zeiss). Average intensity projections were used for analysis. NeuN^+^ neurons in the hippocampus of WT mice were manually counted within regions of interest (ROI) of the same size and comparable localization between mice. Counterstaining with DAPI was used to identify and separate neuronal cell bodies. Synapsin^+^ puncta in the ventral horn were quantified by a customized counting mask in ImageJ. The ROI of the motor neurons for the integrin αV/β analysis was manually determined. Mean fluorescence intensity (MFI) of irisin was calculated by using Fiji (ImageJ, NIH) with background subtraction. NeuN or GFAP immunofluorescence was used to define ROIs corresponding to neuronal cells or astrocytes. NeuN^+^ or GFAP^+^ ROIs were further filtered to include only those overlapping with DAPI^+^ nuclear masks, and the mean irisin intensity was measured within these DAPI-confirmed NeuN^+^ or GFAP^+^ ROIs (Supplementary Data 6). Analysis was standardized across conditions and performed to respective controls. For quantification, the hippocampus was divided into up to two sections per animal and the spinal cord into up to four sections per animal.

Eyes were postfixed in 4% PFA for 4 h and afterwards transferred to 30% sucrose (in PBS) for cryoprotection. Retinas were dissected from the eyes and four even cuts were made to create a flat retina, which was then processed for RGC staining. Retina samples were then stained with Anti-BRN3A antibody^47^. Briefly, optic nerves were dissected and evenly divided into three parts, vertically placed into Tissue-Tek mould (10 mm × 10 mm × 5 mm) filled with O.C.T., snap-frozen directly on dry ice, cross-sectioned at 16-μm thickness and then mounted on slides for further immunofluorescence staining with IBA1 and GFAP. After three washes with PBS, the slices were incubated with fluorophore-conjugated secondary antibody in the dark for 2.5 h, washed with PBS three times and then mounted in ROTIMount FluorCare DAPI (Carl Roth, cat. no. HP20.1)^47^. Fluorescent images of the retina or optic nerve were acquired with Zeiss Axio Observer Z1. Constant exposure settings were used between samples. In each retina section, 12 regions (central, middle and peripheral; four locations each) were selected and the number of BRN3A^+^ RGCs was determined using a semi-automated MATLAB algorithm previously developed^48^. The positive area of IBA1 and GFAP was calculated for proximal, middle and distal regions of optic nerves using Fiji (ImageJ, NIH). Manual thresholding was applied and kept consistent between samples. Each reported value represents the average of quantifications from the three regions within each optic nerve^47^.

### TEM

After dissection and postfixation in 4% PFA and 1% GA in 0.1 M phosphate buffer, cervical spinal cords were cut in 1-mm transverse sections and postfixed in 1% osmium tetroxide (OsO_4_) for 1 h. Following osmication, the sections were dehydrated using ascending steps of ethyl alcohol concentration, followed by two rinses in propylene oxide. Infiltration of the embedding medium was performed by immersing the pieces in a 1:1 mixture of propylene oxide and Epon and finally in neat Epon and hardened at 60 °C. Semithin sections (0.5 µm) were prepared for light microscopy, mounted on glass slides and stained for 1 min with 1% Toluidine blue. Ultrathin sections (60 nm) were cut and mounted on copper grids. Sections were stained using uranyl acetate and lead citrate. Thin sections were examined, and motoneurons from the anterior horn were photographed using an EM900 (Zeiss) electron microscope equipped with a TRS 2K digital camera (A. Trondle, Germany). Panoramic overview images of single motoneurons were obtained by tiling 3 × 3 TEM images obtained at a magnification of 7,000×. Five motor neurons per animal were analysed for synapses. Numbers of synapses and synaptic contacts were quantified. A synaptic contact with an active zone was defined as a postsynaptic density on the motor neuron connected to a presynapse with visible vesicles. For each motor neuron, we additionally quantified the percentage of cell membrane covered by synaptic contacts. Ten motor neurons per animal were analysed for mitochondrial density and elongation. ROIs of the same size were defined within the cytoplasm of the motor neuron, and the mitochondria within the ROI were counted. The mitochondria count was used to calculate mitochondrial density per area of the ROI. Furthermore, the mean mitochondrial size and the elongation (aspect ratio) were calculated. The analysis was performed using ImageJ (NIH).

### Antibodies

All primary and secondary antibodies, with source, dilutions and validations, are presented in tables in the Supplementary Information.

### Nucleus isolation and flow cytometric sorting of the spinal cord

After the mice were perfused with ice-cold PBS, the spinal cords were dissected out into ice-cold HBSS and snap-frozen in liquid nitrogen until nuclei isolation. The spinal cord was homogenized with potters (25× loose, 20× tight) in 2 ml EZ lysis buffer (10 mM Tris pH 7.4, 10 mM NaCl, 5 mM MgCl_2_, 0.5% NP-40) with Recombinant RNase Inhibitor (Takara Bio 2313 A). The preparation was spun down at 500*g* for 4 min at 4 °C to remove debris. The nuclei suspension was resuspended in 1,000 μl ice-cold nucleus incubation buffer (340 mM sucrose, 2 mM MgCl_2_, 25 mM KCl, 65 mM glycerolphosphate, 5% glycerol, 1% EDTA, 2% BSA) with P1000 with RNAse-free filter tips. The nuclei suspension is transferred through a 30-μM filter (preseparation filter for 15 ml falcon) on top of 15 ml falcon. The suspension was then stained with anti-NeuN 1:500 (Alexa Fluor 647 tagged anti-NeuN antibody ab190565) and Hoechst 33342 (Invitrogen H3570) with RNAseI inhibitor 15 min before sorting using a BD FACSAria II Cell Sorter (MGH Pathology: Flow Cytometry Core, nozzle 100 µm). NeuN^+^ and NeuN^−^ nuclei fractions were collected in 5 ml PBS with 1% endotoxin-free BSA with RNAseI Inhibitor and snap-frozen.

### qRT–PCR

Total RNA was isolated from the gastrocnemius muscle, liver or sorted nuclei using the RNeasy Mini Kit (Qiagen) according to manufacturer’s instructions. For the gastrocnemius muscle, RNA was reverse transcribed to cDNA with the RevertAid H Minus First Strand cDNA Synthesis Kit (Thermo Fisher). Gene expression was analysed by qPCR with reverse transcription (qRT–PCR) performed in an ABI Prism 7900 HT Fast Real-Time PCR System (Applied Biosystems) using TaqMan Gene Expression Assays (Thermo Fisher) for *Ppargc1a* (Mm00464452_m1), *Fndc5* (Mm01181543_m1) and *Tbp*(Mm01277042_m1). Gene expression was calculated as 2^−ΔCT^ relative to *Tbp* as endogenous control. qRT–PCR was performed in triplicates using Power SYBR Green PCR Master Mix (4367660, Thermo Fisher Scientific) in a QuantStudio5 Real-Time PCR system (Applied Biosystems). For liver tissue, first-strand cDNA was generated using equal amounts of RNA and the High Capacity cDNA Reverse Transcription Kit with RNase Inhibitor (4374967, Thermo Fisher Scientific). For sorted nuclei from the spinal cord, first-strand cDNA was generated using equal amounts of RNA and the Superscript IV first-strand synthesis system (18091050, Invitrogen, Thermo Fisher Scientific). qRT–PCR was performed using Power SYBR Green PCR Master Mix (4367660, Thermo Fisher Scientific) in a QuantStudio5 Real-Time PCR system (Applied Biosystems). Relative quantification of gene expression normalized to *Rps18* (liver) or *Tbp* (sorted nuclei) was determined by the comparative Ct method (ΔΔCt). Primers were custom designed and ordered from Integrated DNA Technologies. Primer sequences are presented in the Supplementary Information as Supplementary Table 5.

### RNA-seq and data analysis

RNA extraction, library preparation and sequencing reactions from fluorescence-activated cell-sorted nuclei were conducted at GENEWIZ. Trimmomatic v.0.36 was used to trim sequence reads, removing any adaptor sequences and low-quality nucleotides. The reads were aligned to the Ensembl mouse reference genome (GRCm38) using STAR v.2.5.2b. with default parameters. The overlap with annotated gene loci was quantified using featureCounts v.1.5.2. Differential expression analysis was performed with DESeq2 (v.3.12) with a two-sided Wald test followed by Bonferroni–Hochberg correction. Genes were called differentially expressed with a minimal log_2_(FC) of |1| and false discovery rate (FDR)-adjusted *P* < 0.05. Gene lists were annotated using biomaRt (v.4.0). Principle component analysis was executed using the scikit-learn package (v. 1.5.1). GSEA was conducted with the clusterProfiler package (v. 4.12.5) and the similarity between gene sets was calculated using the Jaccard correlation coefficient (Supplementary Data 6). Data processing was performed on a local machine with the same computational environment settings.

### Data integration with scRNA-seq data from voluntary free-wheel running mice

In Sun et al., young (2 months old) and old (16-months old) male C57BL/6J mice underwent a 12-month voluntary running wheel regimen, spinal cord tissues were collected and snRNA-seq was performed (CRA007207)^33^. The publicly available DEG list was filtered for: spinal cord tissue, neuronal cell type and young and old running animals. DEGs were defined as genes with adjusted *P* value <0.05. These DEGs were subsequently compared with our DEG (data reported in Supplementary Data 3).

### Data integration with motor neuron BAC-TRAP data from EAE mice

In Schattling et al., RNA from spinal cord motor neurons from EAE and healthy control mice were collected during the acute phase using BAC-TRAP technology and bulk RNA-seq was performed (GSE104899)^27^. In Woo et al., RNA from spinal cord NeuN^+^ nuclei from EAE and healthy control mice was analysed using bulk RNA-seq (GSE249192)^29^. EAE-dysregulated genes were defined as genes with a minimal log_2(_FC) <−1 and FDR-adjusted *P* < 0.05 in both datasets. These DEGs were subsequently compared with our DEG list results (data reported in Supplementary Data 4 and 5).

### Analysis of published EAE sequencing datasets to identify cell type-specific EAE-regulated genes

Raw count matrices and metadata from publicly available RNA-seq datasets were retrieved from the Gene Expression Omnibus (GEO) repository. GSE118948 contains a single-cell sequencing dataset of CD45^+^ leucocytes in EAE isolated from the spinal cords of EAE mice 15 days after immunization. Analysis of the single-cell data was performed using Seurat. The provided cell type annotation from the original publication^24^ was used to perform Wilcoxon-test differential expression analysis of T cells, neutrophils, dendritic cells, macrophages, monocytes, CNS-associated monocytes and microglia of healthy and acute EAE mice. Bulk sequencing data from GSE194071 (ref. ^25^) were used to analyse spinal cord microglia of acute and chronic recovery EAE mice, GSE100329 (ref. ^26^) was used to analyse spinal cord astrocytes from acute and chronic progressive EAE mice, GSE104899 (ref. ^27^) and GSE279707 (ref. ^29^) were used to analyse spinal cord motor neurons from acute EAE mice and GSE249192 (ref. ^28^) was used to analyse cortical motor neurons from acute and chronic EAE mice. Bulk sequencing data were analysed using DESeq2. We calculated the signed −log_10_ FDR-adjusted *P* value for the visualization. The methodological details for the stimulations, treatments or preparations can be found in the respective references.

### Marker analysis

Published marker genes for reactive astrocytes^31^, disease-associated astrocytes^30^ and activated microglia^32^ were compared with our GFP versus treatment differential expression data from spinal cord NeuN^−^ nuclei. Genes were visualized using log_2_(fold change, FC) and adjusted *P* values from our RNA-seq analysis.

### Statistical analyses

The complex bioinformatic analyses of the RNA-seq data are described above in detail. Statistical analysis of the remaining data was performed using GraphPad Prism 10.3.1. Significance was assigned to differences with a *P* value less than 0.05 unless stated otherwise. Data are expressed as mean ± s.e.m. unless stated otherwise. Sample size was based on previously published studies using similar methodologies. Statistical analyses were performed using the appropriate test indicated in the figure legends. To determine the appropriate sample size for the *Fndc5*-KO EAE exercise experiment, we performed a power analysis (GPower 3.1.9.7. software)^49^ on the basis of the effect size and variance measured in the WT experiments. Using the standard *α* error of 0.05 and a power of 0.80, the required sample size is seven animals to detect a significant difference between running and sedentary animals. For most analyses, animals were considered the biological replicates. For single-cell electron microscopy imaging analyses of synapses, mitochondria or confocal imaging analysis of irisin binding on individual cells, we considered the individual cells as the biological replicate, as it is well documented that phenotypic heterogeneity exists between cell populations in the same tissue owing to variance in the local microenvironment (reviewed in Mattiazzi Usaj et al.)^50^. Figures show biological replicates. Outlier identification was first performed via ROUT (*Q* = 0.2%). The Shapiro–Wilk test was used to analyse normality. In normally distributed data, differences between two experimental groups were determined using an unpaired, two-tailed Student’s *t*-test with Bonferroni correction where applicable; in non-normally distributed data, differences between two experimental groups were determined using an unpaired, two-tailed Mann–Whitney test. Statistical analysis of the clinical scores in the EAE experiments was performed by applying a Mann–Whitney *U* test to the recovery score (maximum score − final score) for each animal. Only mice that had a minimum EAE score of 1 were included in these analyses. Missing values in the statistical analysis of EAE clinical scores (chronic EAE cohorts) were handled by conservative mean imputation. Specifically, for each missing daily score, we replaced the value with the mean clinical score of the corresponding experimental group on that same day. This approach ensured that the imputed values did not bias the analyses towards higher or lower disease severity. In total, three animals required imputation: one in the *Fndc5*-KO cohort, one in the WT voluntary wheel-running cohort, and one in the AAV8–irisin cohort. For the AAV8–irisin cohort, only AAV8–irisin–Flag mice with a minimum increase of 5% irisin expression compared with AAV8–GFP control animals were included. For the WT running and AAV8–irisin EAE, data were pooled from two separate experiments. qRT–PCR mRNA expression levels of irisin in the liver and irisin plasma levels are only shown for animals analysed for EAE. Data for the MFI analysis of irisin in NeuN^+^ ROI and GFAP^+^ ROI were pooled from two separate experiments, and data were normalized to the respective healthy control. Significant results are indicated with an asterisk: *P < 0.05, **P < 0.01, ***P < 0.001, ****P < 0.0001. For CFC, exclusion criteria were pre-established following published guidance^51^. Mice that did not freeze after receiving the shocks on day 1 were excluded from that analysis, as we cannot use freezing as a proxy for learning or memory. One-way analysis of variance (ANOVA) with uncorrected Fisher’s least significant difference (LSD) was used for analysis. For all experiments, all stated replicates are biological replicates. Two-way ANOVA with Šídák’s multiple comparisons test or repeated-measures-two-way ANOVA with Tukey’s post hoc test were used. Operators were blinded to the true experimental groups during data collection and image analysis by de-identifying all samples with generic unique IDs.

### Reporting summary

Further information on research design is available in the Nature Portfolio Reporting Summary linked to this article.

## Supplementary information

Supplementary Information Supplementary Tables 1–5.

Reporting Summary

Supplementary Data 1 Complete dataset of flow cytometry of peripheral blood and the details of the statistical analysis.

Supplementary Data 2 Complete dataset of flow cytometry of spinal cord and the details of the statistical analysis.

Supplementary Data 3 Overlap analysis with Sun dataset.

Supplementary Data 4 Overlap analysis with Schattling dataset.

Supplementary Data 5 Overlap analysis with Woo dataset.

Supplementary Data 6 Jupyter Notebook with relevant code.

## Source data

Source Data Fig. 1 Numerical source data and statistical details for Fig. 1.

Source Data Fig. 2 Numerical source data and statistical details for Fig. 2.

Source Data Fig. 3 Numerical source data and statistical details for Fig. 3.

Source Data Fig. 5 Numerical source data and statistical details for Fig. 5.

Source Data Fig. 6 Numerical source data and statistical details for Fig. 6.

Source Data Extended Data Fig. 1 Numerical source data and statistical details for Extended Data Fig. 1.

Source Data Extended Data Fig. 2 Numerical source data and statistical details for Extended Data Fig. 2.

Source Data Extended Data Fig. 3 Numerical source data and statistical details for Extended Data Fig. 3.

Source Data Extended Data Fig. 4 Numerical source data and statistical details for Extended Data Fig. 4.

Source Data Extended Data Fig. 5 Numerical source data and statistical details for Extended Data Fig. 5.

Source Data Extended Data Fig. 6 Numerical source data and statistical details for Extended Data Fig. 6.

## Data Availability

The RNA-seq dataset generated here is available at the Gene Expression Omnibus (GEO) repository under accession number GSE282166. The following published datasets were used: GSE104899, GSE118948, GSE194071, GSE100329, GSE279707, GSE249192, and CRA007207 from the Genome Sequence Archive (GSA). Source data are provided with this paper.

## Code availability

The relevant code is provided in Supplementary Data 6.
