# Supplementary material for: The exercise hormone irisin has neuroprotective effects in a mouse model of multiple sclerosis
Source: Nat Metab. 2026 May 21;8(5):1051–66. doi: 10.1038/s42255-026-01527-7 (PMC13218937; doi:10.1038/s42255-026-01527-7)
Supplement: Supplementary file 1 — Supplementary Tables 1–5. [file 42255_2026_1527_MOESM1_ESM.pdf]

nature metabolism

Supplementary information https://doi.org/10.1038/s42255-026-01527-7

The exercise hormone irisin has neuroprotective effects in a mouse model of multiple sclerosis

In the format provided by the authors and unedited

Table S1. List of primary antibodies

Antibody	Company/Origin	Catalog number	Isotype	Application	Dilution	Clone	Lot	Citation
Chicken Anti-NeuN	Millipore	ABN91	IgY	IF-spinal cord	1:500	Polyclonal	1759025	Black et al., *Front Cell Neurosci*, 2017
Chicken Anti-NeuN	Millipore	ABN91	IgY	IF-spinal cord	1:500	Polyclonal	4073585	Black et al., *Front Cell Neurosci*, 2017
Mouse Anti-GFAP	Merck	MAB3402	IgG	IHC-spinal cord	1:200	Monoclonal	3984892	Orr et al, *Nat Neurosci*, 2015
Rabbit Anti-IBA1	Wako	019-19741	N/A	IHC-spinal cord	1:500	Polyclonal	LEN4378	Erny et al, *Nat Neurosci*, 2015
Rabbit Anti-IBA1	Wako	019-19741	N/A	IF-optic nerve	1:1000	Polyclonal	2186435	Choi et al., *Science*, 2018
Rabbit Anti-CD3	Abcam	AB16669	N/A	IHC-spinal cord	1:50	Monoclonal	1027923-15	Elhai et al, *Nat. Commun*, 2023
Guinea Pig Anti-Synapsin 1/2	Synaptic Systems	106 004	N/A	IF-spinal cord	1:200	Polyclonal	1786359 and 3-34	Lagache T et al., *Nat Commun*, 2018
Rabbit Anti-Brn3a	Synaptic Systems	411 003	N/A	IF-retina	1:1000	Polyclonal	1-11	Gharagozloo et al., *Acta Neuropathol*, 2021
Chicken Anti-GFAP	Thermo Fisher	PA1-10004	IgY	IF-optic nerve	1:2000	Polyclonal	ZH444313 1	Zhao et al., *Mol Neurodegener*, 2022
Rabbit Anti-NeuN	Abcam	Ab190565	IgG	Flow Cytometry	1:500	Monoclonal	4094256	Hahn et al., *Nature* 2023
Mouse Anti-NeuN	Merck	MAB377	IgG	IHC-spinal cord	1:100	Monoclonal	3832727	Paşca et al., *Nat. Methods*, 2015
Mouse anti-irisin	R&D	MAB8880-100	IgG	ELISA	4ug/mL	Monoclonal	CKBN012211 1	Islam et al., *Nat Metabolism*, 2021
Mouse Anti-irisin	R&D	MAB8880-100	IgG1	IF-spinal cord	1:600	Monoclonal	CKBN012403 1	Islam et al., *Nat Metabolism*, 2021

Rabbit anti-DYKDDDDK tag	Cell Signaling	14793S	IgG	ELISA	1:1000	Monoclonal	7	Islam et al., Nat Metabolism, 2021
Rabbit Anti-Chat	Thermo Fisher	PA5-29653	IgG	IHC-spinal cord	1:500	Polyclonal	VD29744 70	Bieniussa et al., *Front Neurol.*, 2022
Rat Anti-MBP	Merck	MAB386	IgG	IHC-spinal cord	1:100	Monoclonal	0702051748	Lodato et al., *Nat Neurosci*, 2014
Mouse Anti-Integrin αVβ5	Merck	MAB1961	IgG1	IF-spinal cord	1:50	Monoclonal	3857728	Roth et al., *Brain*, 2013
Chicken Anti-GFAP	Abcam	Ab4674	IgG	IF-spinal cord	1:500	Polyclonal	1056567-1	*Cebrian-Silla, elife, 2021*

Table S2. List of Secondary Antibodies

Antibody	Company/ Origin	Catalog number	Isotype	Application	Dilution	Lot
Donkey anti-chicken Cy3	Jackson Immuno Research	703-165-155	IgG	IF	1:500	
Donkey anti-guinea pig 488	Jackson Immuno Research	706-545-148	IgG	IF	1:500	131203
Donkey Anti Chicken Alexa 488	Jackson Immuno Research	703-545-155	IgY	IF	1:600	
Donkey Anti Rabbit Alexa 555	Abcam	Ab150074	IgG	IF	1:600	
Goat Anti Guinea Pig Alexa 647	Abcam	Ab150187	IgG	IF	1:600	
Goat anti-Chicken Alexa-488	Invitrogen	A11039	IgY	IF	1:1000	2566343
Goat-anti-rabbit-Alexa 594	Invitrogen	A11012	IgG	IF	1:1000	2307236
Donkey Anti Mouse 647	Abcam	Ab150111	IgG	IF	1:500	10441582
Fab fragment donkey anti-mouse IgG	Jackson Immuno Research	715-007-003	IgG	IF	1:200	167037
Goat anti-rabbit 555	Invitrogen	A21429	IgG	IF	1:500	3137323
Goat anti-mouse 555	Invitrogen	A21424	IgG	IF	1:500	2752677
Goat anti-chicken 647	Invitrogen	A21449	IgG	IF	1:500	2079903

Table S3. Flow Cytometry Panel for Longitudinal Blood Immunophenotyping.

Fluor	Marker	Titered concentration (µl/50µl)
Surface markers		
BUV395	CD69	0.5
BUV563	CD8a	1
BUV737	CD11b	0.5
BUV805	IgM	0.75
BV421	CD25	1
BV480	IgD	0.5
BV510	CD138	1
BV570	Ly-6C	0.5
BV605	PD-L1	0.5
BV650	NK 1.1	0.5
BV711	CD3	1
BV785	CD11c	0.5
FITC	CD74	1
PerCP	Ly-6G	0.5
PerCP/Cy5.5	CD86	1
PE	CD39	1
PE/Cy5	CD19	0.375
PE/Cy7	CD80	1
Alexa Fluor 647	CD68	0.5
APC/Cy5.5	CD4	0.5
Alexa Fluor 700	B220	0.5
APC/Cy7	CD45	0.5
Intracellular markers		
PE-eFluor 610	FOXP3	1

Table S4. Flow Cytometry Panel for Spinal Cord Immunophenotyping.

Fluor	Marker	Titered concentration (µl/50µl)
Surface markers		
BUV395	CD69	0.5
BUV496	NK 1.1	0.75
BUV563	CD8a	1
BUV661	CD80	1.25
BV421	CD25	1
Pacific Blue	CD11b	0.25
BV510	CD138	1
BV570	Ly-6C	0.5
BV711	CD3	1
BV785	CD11c	0.5
BB515	CD206	1.25
Alexa Fluor 488	P2RY12	1
PerCP	Ly-6G	0.5
PE/Cy5	CD19	0.5
Alexa Fluor 647	CD68	0.5
APC/Cy5.5	CD4	0.5
Alexa Fluor 700	B220	0.5
Zombie NIR	Zombie NIR	
APC/Cy7	CD45	0.5
Intracellular markers		
BUV737	IFN-Gamma	1
BV605	IL-4	1
BV650	TNF-alpha	0.75
Alexa Fluor 532	IL-27/35	1
PerCP/Cy5.5	IL-2	1
PerCP-eFluor 710	IL-1b	0.75
PE	TGF Beta (LAP)	1.5
PE-eFluor 610	FOXP3	1
PE/Cy7	IL-10	1
APC	IL-6	1

Table S5. List of primers

Primer name	Forward primer sequence	Reverse primer sequence
Gfap	ATTGCTGGAGGGCGAAGAAA	CTTTTGCCCCCTCGGATCT
Rbfox3	CCCATATGCCAATGGCTGGA	GGGTAGGGGAAACTGGTCAC
Gad65	TGGGAAGCCTCAACACACAA	TAATCACTGGCGCCACCTTT
Olig2	CACAGGAGGGACTGTGTCCT	GAGGAGGTGCTGGAGGAAG
Plp1	CCCGACAAGTTTGTGGGCAT	GTACACAGGTACAGCCGAGC
Slc1a3	TCACGTCCTTGCGTGTCAGTGTC	CCCGGGGTGTCTTCCTCCCAT
Tmem119	GTGTCTAACAGGCCCCAGAA	AGCCACGTGGTATCAAGGAG
Cx3cr1	TCACCGTCATCAGCATCGAC	TGCACTGTCCGGTTGTTCAT
Rbfox3	CCCATATGCCAATGGCTGGA	GGGTAGGGGAAACTGGTCAC
Fndc5_nt	CTGGAGGATGAAGTGGTCATTG	TGGTGTTCACCTCCTGAATG
Rps18	CCTCACGCAGCTTGTTGTCTA	CATGCAGAACCCACG ACAGTA
Tbp	ATCTACCGTGAATCTTGGCTGT	CTGCAGCAAATCGCTTGGG
