# Supplementary material for: The exercise hormone irisin has neuroprotective effects in a mouse model of multiple sclerosis
Source: Nat Metab. 2026 May 21;8(5):1051–66. doi: 10.1038/s42255-026-01527-7 (PMC13218937; doi:10.1038/s42255-026-01527-7)
Supplement: Supplementary file 2 — Reporting Summary [file 42255_2026_1527_MOESM2_ESM.pdf]

nature portfolio

Corresponding author(s): Christiane Wrann

Last updated by author(s): Dec 4, 2025

Reporting Summary

Nature Portfolio wishes to improve the reproducibility of the work that we publish. This form provides structure for consistency and transparency in reporting. For further information on Nature Portfolio policies, see our Editorial Policies and the Editorial Policy Checklist.

Statistics

For all statistical analyses, confirm that the following items are present in the figure legend, table legend, main text, or Methods section.

n/a	Confirmed	
☐	☒	The exact sample size (n) for each experimental group/condition, given as a discrete number and unit of measurement
☐	☒	A statement on whether measurements were taken from distinct samples or whether the same sample was measured repeatedly
☐	☒	The statistical test(s) used AND whether they are one- or two-sided<br>*Only common tests should be described solely by name; describe more complex techniques in the Methods section.*
☐	☒	A description of all covariates tested
☐	☒	A description of any assumptions or corrections, such as tests of normality and adjustment for multiple comparisons
☐	☒	A full description of the statistical parameters including central tendency (e.g. means) or other basic estimates (e.g. regression coefficient) AND variation (e.g. standard deviation) or associated estimates of uncertainty (e.g. confidence intervals)
☐	☒	For null hypothesis testing, the test statistic (e.g. F, t, r) with confidence intervals, effect sizes, degrees of freedom and P value noted<br>*Give P values as exact values whenever suitable.*
☒	☐	For Bayesian analysis, information on the choice of priors and Markov chain Monte Carlo settings
☒	☐	For hierarchical and complex designs, identification of the appropriate level for tests and full reporting of outcomes
☐	☒	Estimates of effect sizes (e.g. Cohen's d, Pearson's r), indicating how they were calculated

Our web collection on statistics for biologists contains articles on many of the points above.

Software and code

Policy information about availability of computer code

Data collection	LSM 900 Carl Zeiss (Germany), Zeiss Axio Observer.Z1, Aurora spectral flow cytometer (Cytek),BD FACSAria II Cell Sorter , Ventana BenchmarXT (Roche, Switzerland, Zeiss MIRAX MIDI Slide Scanner (Carl Zeiss, MicroImaging GmbH, Germany), EM900 (Zeiss) electron microscope equipped with a TRS 2K digital camera (A. Trondle, Moorenweis, Germany), ANY-maze software v6.0 (Stoelting Co. Wood Dale, IL), VitalView Animal Activity v1.4 (Starr life science Corp, Oakmont, PA)
Data analysis	SpectroFlo® software (v. 3.3.0), FlowJo software, (v. 10.8.1) (TreeStar, Inc.), ImageJ (v. 1.0), QuPath 0.30 software, GraphPad (v. 10.3.1), Trimmomatic (v. 0.36), STAR (v. 2.5.2b), featureCounts (v. 1.5.2), DESeq2 (v.3.12), biomaRt (v.4.0), scikit-learn package (v. 1.5.1), clusterProfiler package (v. 4.12.5), GPower (v. 3.1.9.7). The relevant code is part of the supplementary data.

For manuscripts utilizing custom algorithms or software that are central to the research but not yet described in published literature, software must be made available to editors and reviewers. We strongly encourage code deposition in a community repository (e.g. GitHub). See the Nature Portfolio guidelines for submitting code & software for further information.

Data

Policy information about availability of data

All manuscripts must include a data availability statement. This statement should provide the following information, where applicable:

- Accession codes, unique identifiers, or web links for publicly available datasets
- A description of any restrictions on data availability
- For clinical datasets or third party data, please ensure that the statement adheres to our policy

RNA-seq dataset generated here is available at the Gene Expression Omnibus (GEO) repository under accession number GSE282166.
The following published data sets were used: sc-RNAseq data from exercised mice CRA007207, GSE118948 contains a single-cell sequencing dataset of CD45+ leucocytes in EAE isolated from the spinal cords of EAE mice 15 days after immunization, bulk sequencing data from GSE19407125 was used to analyze spinal cord microglia of acute and chronic recovery EAE mice, GSE10032926 was used to analyze spinal cord astrocytes from acute and chronic progressive EAE mice, and GSE10489927 and GSE27970732 were used to analyze spinal cord motoneurons from acute EAE mice, GSE24919247

Research involving human participants, their data, or biological material

Policy information about studies with human participants or human data. See also policy information about sex, gender (identity/presentation), and sexual orientation and race, ethnicity and racism.

Reporting on sex and gender	N/A
Reporting on race, ethnicity, or other socially relevant groupings	N/A
Population characteristics	N/A
Recruitment	N/A
Ethics oversight	N/A

Note that full information on the approval of the study protocol must also be provided in the manuscript.

Field-specific reporting

Please select the one below that is the best fit for your research. If you are not sure, read the appropriate sections before making your selection.

☒ Life sciences ☐ Behavioural & social sciences ☐ Ecological, evolutionary & environmental sciences

For a reference copy of the document with all sections, see nature.com/documents/nr-reporting-summary-flat.pdf

Life sciences study design

All studies must disclose on these points even when the disclosure is negative.

Sample size	Samples size was based on previously published studies that had sufficient power in methodologies similar to the ones used in our study: Choi SH. et al., Science 2018; Islam, MR. et al., Nature Metabolism, 2021; Zalocusky, KA. et al., Nature Neuroscience, 2021.To determine the appropriate sample size for the Fndc5 KO EAE exercise experiment, a power analysis (GPower 3.1.9.7. software, Faul et al., Behav Res Methods, 2007) based on the effect size and variance measured in the WT experiments was conducted.
Data exclusions	Outliers were identified using the ROUT (Q = 0.2%). The following pre-established exclusion criteria were used: For CFC, mice that did not freeze after receiving the shocks on day 1 were excluded from that analysis as we cannot use freezing as a proxy for learning or memory (Kim et al. Nat. comm. 2020). Only mice that had a minimum EAE score of 1 were included in these analyses. For the irisin experiments, only AAV8-irisin-FLAG mice with a minimum increase of 5% irisin expression compared to AAV8-GFP control animals were included. In the exercise cohorts, mice that did not run a minimum of 1 km/24 hours on average were excluded.
Replication	All experimental findings were reproduced as biological replicates as stated in figure legends. All additional replication attempts were successful.
Randomization	For in vivo studies, mice were randomly assigned to treatment groups.
Blinding	During all experiments, investigators were blinded to experimental conditions. Running conditions could not be blinded due to the wheels in the cages. However, the tissue analysis of this cohort was blinded using unique generic IDs. Data analysis was performed using automated software. During image analysis, experimenters were blinded to group allocation during data collection and data analysis. For all other experiments, operators were blinded to the actual experimental groups during data collection by de-identifying all samples with unique generic IDs. Blinding for data analysis was not necessary since these were objective, quantitative assays not requiring subjective interpretation.

Reporting for specific materials, systems and methods

We require information from authors about some types of materials, experimental systems and methods used in many studies. Here, indicate whether each material, system or method listed is relevant to your study. If you are not sure if a list item applies to your research, read the appropriate section before selecting a response.

Materials & experimental systems

n/a	Involved in the study	
☐	☒	Antibodies
☒	☐	Eukaryotic cell lines
☒	☐	Palaeontology and archaeology
☐	☒	Animals and other organisms
☒	☐	Clinical data
☒	☐	Dual use research of concern
☒	☐	Plants

Methods

n/a	Involved in the study	
☒	☐	ChIP-seq
☐	☒	Flow cytometry
☒	☐	MRI-based neuroimaging

Antibodies

Antibodies used

Chicken Anti-NeuN (Millipore) ABN91, lot 1759025 and 4073585; Mouse Anti-NeuN (Merck), Mab377, lot 3832727; Rabbit anti-NeuN (Abcam), AB190565, lot 4094256; Chicken Anti-GFAP (Abcam) Ab4674, lot 1056567-1; Chicken Anti-GFAP (ThermoFisher) PA1-10004, lot ZH4443131; Mouse anti-GFAP (Merck), MAB3402, lot 3984892, Rabbit Anti-Iba1 (Wako) 019-19741, lot LEN4378 and 2186435; Rabbit anti-CD3 (Abcam), AB16669, lot 1027923-15, Guinea-pig anti-synapsin 1/2 (Synaptic Systems), 106004, lot 1786359 and 3-34; Rabbit anti-Brn3a (Synaptic Systems), 411003, lot 1-11; Mouse anti-irisin (R&D), MAB8880-100, lot CKBN0122111 and CKBN0124031; ; Rabbit anti-DYDDDDK (Cell Signaling), 14793S, lot 7; Rabbit anti-Chat (ThermoFisher), PA5-29653, lot VD2974470; Rat anti-MBP (Merck), MAB386, lot 0702051748, Mouse Anti-Integrin αVβ5 (Merck) MAB1961, lot 3857728

Validation

Validation was performed in-house and with validation citations:
Chicken Anti-NeuN (Millipore): Black, BJ. et al., Front Cell Neurosci, 2017;
Mouse Anti-GFAP (Merck): Orr et al, Nat Neurosci, 2015;
Anti-Iba1, Rabbit (Wako): Choi, SH. et al., Science, 2018, Erny et al, Nat Neurosci, 2015;
Rabbit Anti-CD3 (Abcam): Elhai et al., Nat. Commun, 2023;
Guinea Pig Anti-Synapsin ½ (Synaptic Systems): Lagache T et al., Nat Commun, 2018;
Rabbit Anti-Brn3a (Synaptic Systems): Gharagozloo et al., Acta Neuropathol, 2021;
Chicken Anti-GFAP (ThermoFisher): Zhao et al., Mol Neurodegener. 2022;
Rabbit Anti-NeuN (Abcam): Hahn et al., Nature 2023;
Mouse Anti-NeuN (Merck): Paşca et al., Nat. Methods, 2015;
Mouse anti-irisin (R&D) and Rabbit anti-DYDDDDK (Cell Signaling): Islam et al, Nat Metabolism, 2021;
Rabbit Anti-Chat (ThermoFisher): Bieniussa et al., Front Neurol., 2022
Rat Anti-MBP (Merck): Lodato et al., Nat Neurosci, 2014
Mouse Anti-Integrin αVβ5 (Merck): Roth et al., Brain, 2013
Chicken Anti-GFAP (Abcam): Cebrian-Silla, elife, 2021

Animals and other research organisms

Policy information about studies involving animals; ARRIVE guidelines recommended for reporting animal research, and Sex and Gender in Research

Laboratory animals

C57BL/6J (000664, JAX) were used as wildtype mice. Fndc5fl/fl-targeted (exon 2 and 3) mice were developed by us in collaboration with the Texas A&M Institute for Genomic Medicine using homologous recombination. Global Fndc5 KO mice (F5KO) were generated by crossing Fndc5fl/fl with B6.FVB-Tg(EIIa-cre)C5379Lmgd/J mice (003724, JAX). All experimental animals were housed in the specific pathogen-free environment animal facility at MGH with a regular 12h light and 12h dark cycle from 07:00 to 19:00, at 20-22°C and 30-70% humidity. All procedures were performed during the light cycle. Mice had free access to water and standard chow (Prolab® IsoPro® RMH 3000, or Altromin 1310, Irradiated). Mice were group-housed except for running experiments. For the wildtype exercise experiments, 6-8-week-old male mice were used. For all other experiments 7-13-week-old female mice were used. Mice were single-housed for the exercise experiments to assess the individual running distance and group-housed for the other experiments. Assignment to experimental groups was random. Tissues were collected at the indicated time points. All procedures were carried out in accordance with the ARRIVE guidelines.

Wild animals

No wild animal was involved in this study.

Reporting on sex

We considered sex as a biological variable in our study design and adhered to the 3R principles (Replacement, Reduction and Refinement). As our previous work has extensively reported exercise effects in females, we selected male animals for the current mechanistic investigations to avoid redundancy. For the therapeutic approach with irisin in the EAE model, we used females only. This decision was made to align with the standard methodological practice in the EAE literature, where therapeutic interventions are predominantly evaluated in females.

Field-collected samples	This study did not involve samples collected from the field.
Ethics oversight	All animal procedures were approved by the Institutional Animal Care and Use Committee (IACUC) of the Massachusetts General Hospital (MGH) or the State Authority of Hamburg, Germany.

Note that full information on the approval of the study protocol must also be provided in the manuscript.

Plants

Seed stocks	N/A, see checklist above
Novel plant genotypes	N/A, see checklist above
Authentication	N/A, see checklist above

Flow Cytometry

Plots

Confirm that:

☒ The axis labels state the marker and fluorochrome used (e.g. CD4-FITC).

☒ The axis scales are clearly visible. Include numbers along axes only for bottom left plot of group (a 'group' is an analysis of identical markers).

☒ All plots are contour plots with outliers or pseudocolor plots.

☒ A numerical value for number of cells or percentage (with statistics) is provided.

Methodology

Sample preparation	Cryopreserved blood samples were washed and resuspended in PBS and stained using a Zombie NIR fixable dye (Biolegend) for 30 min. The cells were then washed and resuspended in PBS containing 1% FBS, 0.01% sodium azide (RICCA Chemical) and 5% FcR blocking reagent (Miltenyi Biotec) for 10 min. Blocked cells were incubated for 30 min with fluorophore-conjugated primary surface antibodies (see Table S3). Surface-stained cells were washed and resuspended in fixation buffer (Biolegend) for 20 min, followed by permeabilization wash buffer (1X) (Biolegend). Permeabilized cells were incubated for 30 min with intracellular antibodies (Table S3). All incubation steps were performed at 4°C, protected from light. Cells were analyzed on an Aurora spectral flow cytometer (Cytek) equipped with 355 nm, 405 nm, 488 nm, 561 nm and 640 nm lasers, using SpectroFlo® software. At least 100,000 events were collected from each sample for analysis. Data were analyzed using FlowJo software, version 10.8.1.<br>Spinal cords were rapidly extracted in ice-cold PBS, transferred to microfuge tubes for mechanical trituration, and then enzymatically dissociated for 25 min at 37°C, using a papain-based enzymatic digestion method according to the manufacturer's specifications (Miltenyi Biotec, Bergisch Gladbach, Germany). Prior to immunolabeling, myelin was removed from cell suspensions using commercially available myelin depletion beads (Miltenyi Biotec). Resulting cell suspensions were labeled for flow cytometry as described above, with the antibodies listed in Table S4. Samples were analyzed by spectral flow cytometry as described above, and at least 1,000,000 events were collected from each sample for analysis.
Instrument	5-Laser Cytek Aurora with Plate Loader
Software	Data was collected using SpectroFlo software (Cytek); data was visualized and analyzed using FlowJo version 10
Cell population abundance	Samples were not sorted, all cells were collected for analysis.
Gating strategy	The gating strategy is illustrated in Extended Data Figure 3. All events were first visualized on forward vs side scatter (FSC/SSC) to ensure that sufficient cells are collected. All events were evaluated on FSC -height vs FSC -area and events for which these parameters scaled proportionally, located on the diagonal were selected as singlets. Very small events, under 1M on the FSC axis, were considered debris and discarded. Singlets were then gated on a live/dead parameter. Positive signal for our viability stain was at 10^6, while the negative control was at 10^4. The live gate was thus considered conservatively below 10^4. Live cells were gated on CD45, and events were considered positive if their intensity was above 10^4. In spinal cord samples, clear populations of CD45-mid/positive and CD45-high cells could be observed. CD45-mi/positive were gated and confirmed to be microglia, by co-expression of P2RY12 and CD68. The CD45-hi cells represent populations of infiltrating peripheral leukocytes, and were further gated. CD3 and CD11b were used to identify CD3-positive/CD11b-negative lymphocytes, and these were further gated on CD3 and NK1.1 to identify CD3-positive/NK1.1.-positive natural killer T cells. The NK1.1-negative cells were gated on CD4 and CD8 to identify CD4-postive T cells and CD8-positive T cells. The CD4-positive T cells were further assessed on CD25 and FOXP3 and double-positive cells were considered regulatory T cells. CD3-negative cells were assessed on CD11b and Ly6G, and double-positive cells were considered granulocytes, likely neutrophils.

Ly6G-negative cells were further separated on CD19 and CD138, and CD19-positive cells were gated as B cells. The remaining CD19-negative cells were further separated by CD11b expression, with a gate set at 10^4. CD11b-positive cells were assessed for NK1.1 and CD11b-positive NK1.1-positive were considered natural killer cells. The remaining CD11b-positive NK1.1-negative myeloid cells were separated on Ly6C and conservatively gated at 10^5 into Ly6C-high classical monocytes/ macrophages and Ly6C-medium/low non-classical monocytes/macrophages. The remaining CD11b-negative cells were again plotted on NK1.1 and CD138, resulting in two distinct populations. The CD138-positive cells were gated as plasma cells. The CD138-negative NK1.1-positive cells were further gated on CD11b and CD11c, and CD11b-negative/low CD11c-positive cells were considered classical dendritic cells.
For blood samples, a slightly modified gating strategy was employed, since fewer cell populations were present in these samples overall. Singlets, live cells and CD45-positive immune cells were identified as above. CD45-positive cells were separated on CD19 and CD3, where CD19-positive cells could be gated as B cells. CD19-negative cells were further separated on CD11b and Ly6G, and double-positive cells were considered neutrophils. Ly6G-negative cells were further separated on CD3 and CD8, identifying CD8-positive T cells. CD8-negative cells were further analyzed on CD3 and CD4, yielding CD4-positive T cells, which were further assessed on CD25 and FOXP3 and double-positive cells were considered regulatory T cells. The remaining CD4-negative cells were assessed on CD3 and NK1.1, identifying CD3-positive NK1.1-positive natural killer T cells, as well as CD3-negative NK1.1-positive natural killer cells. The remaining NK1.1-negative cells were assessed on CD3 and CD138, and CD3-negative CD138-positive cells were considered plasma cells. The CD138-negative cells were evaluated on CD68 and CD11b. CD11b-positive cells were assessed on Ly6C and gated at 10^4 into Ly6C-high classical monocytes and Ly6C-medium/low non-classical monocytes. The CD11b-negative CD68-negative cells were further gated on CD11b and CD11c, and CD11b-negative/low CD11c-positive cells were considered classical dendritic cells.
For most markers where the population boundaries were not clearly defined, FMOs were used to determine the appropriate cut-off intensity, or an intensity of 10^4 was considered the boundary for gating, and was kept consistent across samples. Similarly, cytokine gating was performed on one control individual animal, and translated consistently across samples.

☒ Tick this box to confirm that a figure exemplifying the gating strategy is provided in the Supplementary Information.
